# Type 2 deiodinase–dependent surge in thyroid hormone controls muscle stem cell quiescence and self-renewal

**DOI:** 10.1172/JCI194925

**Published:** 2026-03-05

**Authors:** Maria Angela De Stefano, Raffaele Ambrosio, Cristina Luongo, Tommaso Porcelli, Daniela Di Girolamo, Caterina Miro, Monica Dentice, Caterina Missero, Domenico Salvatore

**Affiliations:** 1 Department of Public Health,; 2 Department of Clinical Medicine and Surgery, and; 3 Department of Biology, University of Naples “Federico II,” Naples, Italy.; 4 CEINGE Biotecnologie Avanzate Franco Salvatore, Naples, Italy.

**Keywords:** Endocrinology, Metabolism, Muscle

## Abstract

Stem cells are critical for the homeostasis of adult tissues. Thyroid hormone (TH), whose intracellular concentration is increased by type 2 deiodinase (D2), is involved in many functions, but its role in quiescence is unknown. Here, we show that D2 marks quiescent stem cells in muscle and skin. Genetic D2 depletion in quiescent muscle stem cells triggered their transition from a G_0_ to a G_Alert_-like state. This increased the proliferative potential of the stem cells but impaired their self-renewal capacity, leading to depletion of the stem cell pool and regenerative failure over time. Mechanistically, TH sustained Notch signaling, and active Notch overexpression partially rescued D2 depletion. Transient pharmacological inhibition of D2 accelerated muscle regeneration and skin wound healing by promoting stem cell expansion. In conclusion, we show that D2 is a critical metabolic enzyme in maintaining stem cell quiescence and in regulating self-renewal.

## Abstract 2

Stem cells play a key role in keeping our tissues healthy and functioning properly. In this study, we looked at how thyroid hormone (TH) helps control these cells. Inside cells, an enzyme called D2 increases the amount of active TH. We discovered that D2 is found in stem cells that are in a resting (or “quiet”) state in muscles and skin. When we removed D2 in these resting cells, they became more active and started dividing more—but over time, this caused the stem cell supply to run out and the tissue couldn’t repair itself properly. We also found that TH helps keep another important signal, called Notch, turned on in these cells. Boosting Notch could partially fix the problems caused by losing D2. Interestingly, blocking D2 with a drug made muscles and skin heal faster by encouraging stem cells to grow. Overall, our work shows that D2 is essential for keeping stem cells in balance and helping tissues stay healthy

## Graphical abstract

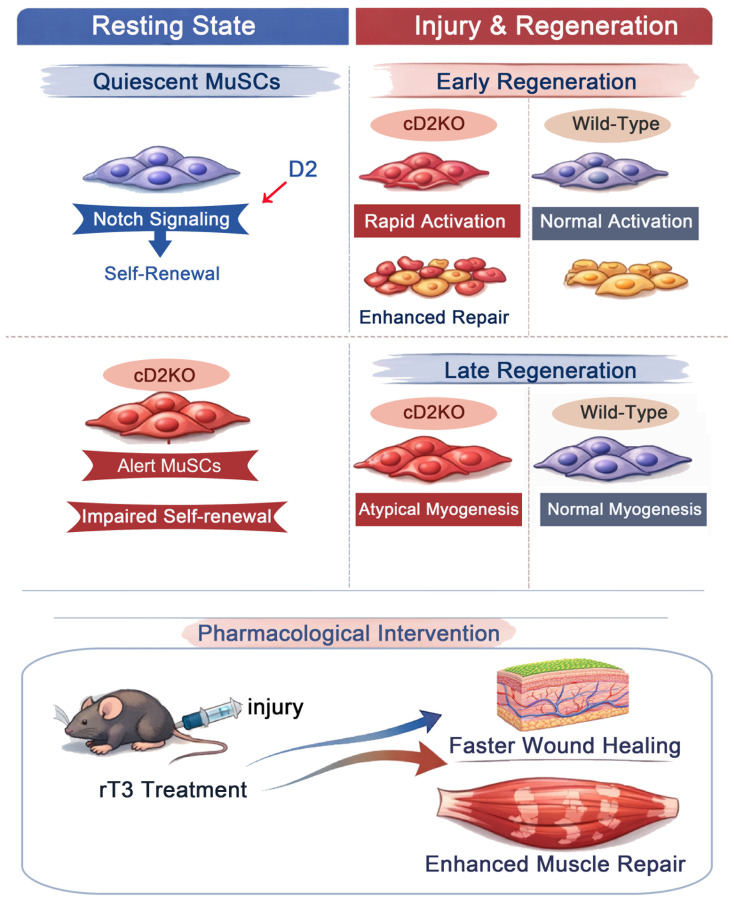

## Introduction

Stem cells sustain tissue homeostasis by generating tissue progeny while self-renewing through cell division (1–3). This process is necessary to ensure continuous tissue maintenance in various organs, and most of the stem cell properties, namely, quiescence, self-renewal, and differentiation, are controlled by the stem cell microenvironment (4–8). Adult resident skeletal muscle stem cells (MuSCs), also known as satellite cells, are normally mitotically quiescent with an extremely low cell turnover but can enter the cell cycle when activated in response to various stresses (9). As an example, upon injury, quiescent MuSCs (qMuSCs) are rapidly activated and generate transient amplifying cells that undergo multiple cell divisions before differentiating to repair the injured muscle (8).

A major advance in this regard was the identification of an intermediate state between the G_0_ (quiescent state) and G_1_ phases of the cell cycle, termed “G_Alert_,” in which MuSCs are rapidly primed to enter the cell cycle in response to injury (10, 11). The G_Alert_ phase is induced by the circulating hepatocyte growth factor activator (HGFA), which is released into the bloodstream from the injured muscle to systemically prime qMuSCs in distant muscles, thereby accelerating tissue repair in the event of a second injury (12).

We previously demonstrated that the intracellular thyroid hormone (TH) concentrations in MuSCs vary dramatically during their transition to the active state, as well as in their subsequent differentiation. These variations depend on the action of the deiodinase enzymes named D2 and D3 (13–17). Specifically, D2 converts the prohormone thyroxine (T4) into the active hormone triiodothyronine (T3), whereas D3 converts T3 into diiodothyronine (T2) and T4 to reverse T3 (rT3), both of which are considered nearly inactive TH metabolites. We have previously shown that a surge in D3 and the consequent reduction in intracellular TH concentration is necessary for the activation and expansion of MuSCs (18). In the later phase of the myogenic process, following MuSC activation, D3 declines while D2 surges, thereby increasing intracellular T3, which is required for proper muscle cell differentiation (18, 19). It is unknown whether and how the regulation of TH action by deiodinases takes place in the context of qMuSCs.

Notably, muscle is not the only tissue in which local TH concentrations are finely tuned during adult life. TH is also an important regulator of skin cell development and homeostasis (20–22). Clinical evidence (23, 24) and studies conducted in hypothyroid rodents (25) suggest that TH is profoundly involved in epidermal proliferation and differentiation, hair growth, and skin wound healing.

The aim of this study was to investigate if and how TH signaling is involved in the control of stem cell quiescence. We identified D2 as a marker of quiescent stem cells in both muscle and skin. Using inducible genetic models to specifically ablate the D2 gene (*Dio2*) in stem cells, we demonstrated that TH-produced D2 maintains the quiescent state in both muscle and skin stem cells and regulates stem cell self-renewal. We provide evidence that in muscle, the maintenance of quiescent state is partially achieved through sustained Notch signaling, which is a master regulator in maintaining the undifferentiated state in quiescence and homeostasis through its action in self-renewal (26–29). In addition, we have shown that D2 depletion induces a transition of qMuSCs from a G_0_ to a G_Alert_-like state, which markedly accelerates their activation and tissue repair upon injury.

Here, we show a pathway by which TH signaling regulates quiescence in 2 relevant stem cell compartments. This process requires a cell-autonomous mechanism capable of increasing intracellular TH levels and adapting them to the specific metabolic needs of quiescent stem cells.

## Results

### D2 marks qMuSCs and preserves their quiescence.

**Figure 1:**
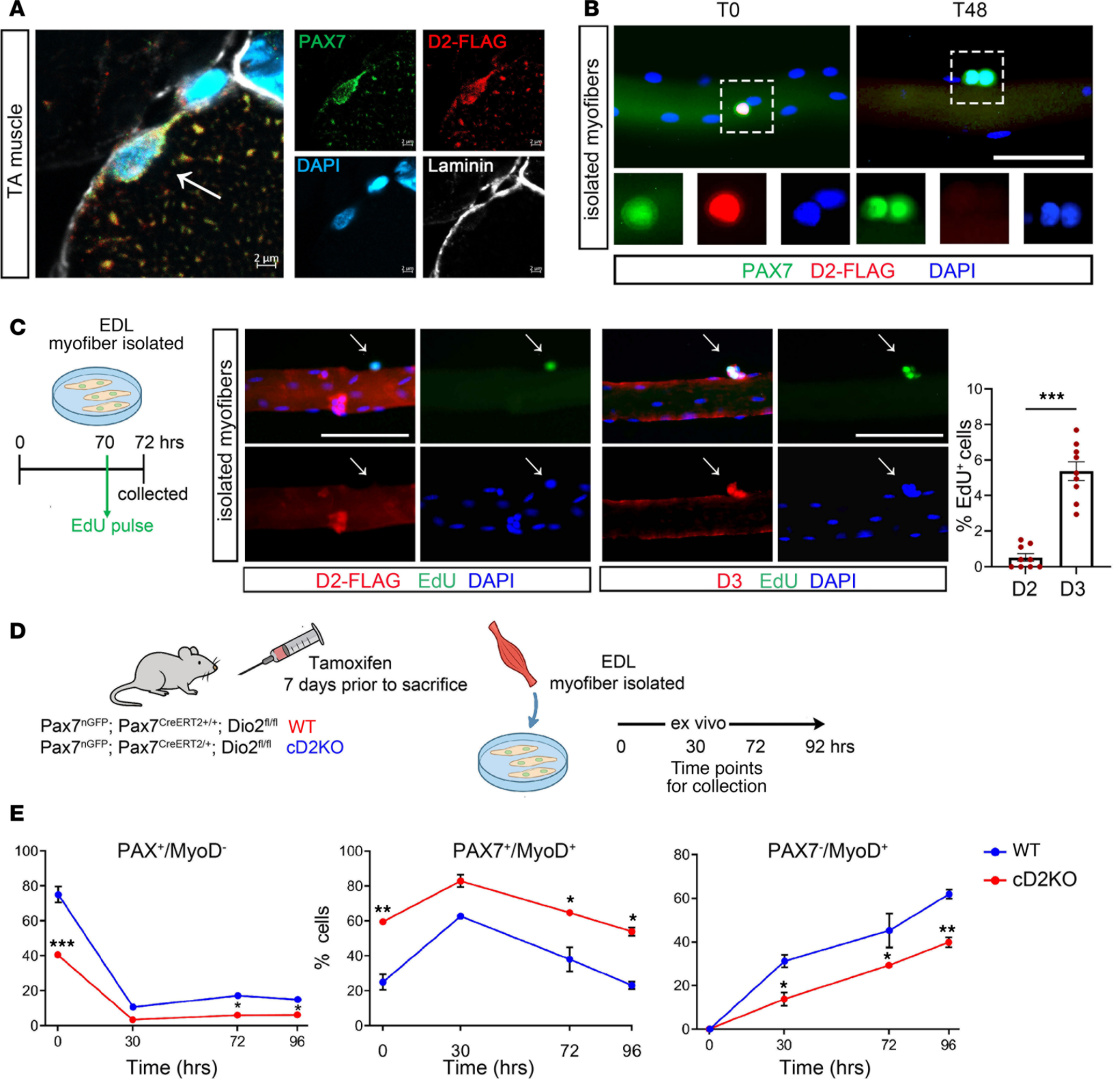
D2 is highly expressed in qMuSCs, and its deletion favors cell activation. (**A**) Representative IF of PAX7, D2, and laminin colocalization. Confocal images of PAX7 (green), D2-FLAG (red), laminin (white) and DAPI (blue) expression on cryosections of TA muscle from D2-FLAG mice. Scale bars: 2 μm. (**B**) Single myofibers with associated MuSCs were immunostained for PAX7 (green) and D2-FLAG (red). Scale bar: 50 μm. T = hours. (**C**) Schematic of the experimental design and representative IF staining for both D2 and D3 versus EdU in cultured fibers at 72 hours. EDL, extensor digitorum longus. Scale bar: 50 μm. At right, quantification of the percentage of D2^+^EdU^+^ and D3^+^EdU^+^ cells. *n* = 9 mice for D2 and *n* = 9 mice for D3. (**D**) Schematic of the experimental design and mouse model used. (**E**) Quantification of the percentage of PAX7^+^MyoD^–^, PAX7^+^MyoD^+^, and PAX7^–^MyoD^+^ cells on isolated myofibers, after in vivo TAM induction, at different time points. *n* = 5 WT and *n* = 4 cD2KO mice. Data are presented as mean ± SEM; *P < 0.05, **P < 0.01, ***P < 0.001 using a Mann-Whitney test when comparing 2 conditions and 2-way ANOVA when comparing multiple conditions.

We analyzed D2 expression within the different subsets of MuSCs (Tg:Pax7-nGFP cells) fractionated into distinct subpopulations based on GFP intensity. D2 expression was highest in Pax7-nGFP^hi^ cells, the most quiescent Pax7 subset (Supplemental Figure 1A; supplemental material available online with this article; https://doi.org/10.1172/JCI194925DS1) (18). Correspondingly, in freshly isolated MuSCs, D2 expression was elevated in quiescent cells and was dramatically reduced upon cell proliferation (Supplemental Figure 1B). Based on these findings, we speculated that D2 might mark qMuSCs. To address this hypothesis, we analyzed D2 expression by immunofluorescence (IF) in resting tibialis anterior (TA) muscle. Using our previously generated 3xFLAG-D2 knockin mouse (30), we found that D2 colocalized with the MuSC marker Pax7 in resting TA muscle (31) (Figure 1A). Interestingly, in isolated myofibers, D2 was highly expressed and colocalized with Pax7^+^ cells immediately after fiber isolation, and its expression declined after 48 hours upon MuSC activation (Figure 1B). Accordingly, D2 was not detectable in proliferating cells identified by EdU incorporation, and its expression was inversely correlated with the deiodinase D3, a metabolic marker of proliferative MuSCs (18) (Figure 1C). Consistent with the role of D2 in enhancing the intracellular TH signaling machinery, we observed that other components of the TH signal machinery — namely, D3 (*Dio3*); the *Thra1*, *Thra2*, and *Thrb* receptors; and the *Mct8*, *Mct10*, and *Oatp1c1* transporters — were expressed in qMuSCs and decreased or increased accordingly as the cells transitioned from a quiescent to an activated state, as evidenced by the expression of the corresponding cell markers (e.g., see *Pax7*, *MyH2*, and *Heyl* in Supplemental Figure 1, C–L).

Given the high expression of D2 in qMuSCs, we hypothesized that D2 may have a role in preserving the quiescent state. To test this, we analyzed the effects of D2 depletion in Tg:Pax7^CreERT2/+^ D2^fl/fl^ mice (cD2KO), in which treatment with tamoxifen (TAM) induced D2 depletion in approximately 80% of qMuSCs (Supplemental Figure 2A). Genetic D2 depletion in vivo before culturing myofibers resulted in a higher percentage of Pax7^+^MyoD^+^ (activated MuSCs) and a lower percentage of Pax7^+^MyoD^–^ (qMuSCs) and Pax7^–^MyoD^+^ cells (differentiating MuSCs) at different time points (Figure 1, D and E). A similar effect was also observed when we blocked D2 in cultured myofibers with rT3 (a specific D2 inhibitor) for 72 hours (Supplemental Figure 2B). After rT3 treatment, we observed a decreased percentage of qMuSCs and an increased percentage of activated MuSCs compared with controls (Supplemental Figure 2, C and D).

Accordingly, in myofibers treated with cytosine β-d-arabinofuranoside (AraC; a chemotherapeutic drug that kills cycling and spares quiescent cells), we detected fewer AraC-resistant Pax7^+^ cells in the absence of D2 compared with WT (Supplemental Figure 2, E–G). This result suggests that D2-depleted MuSCs are more prone to proliferate and that TH locally produced by D2 may be involved in maintaining stem cell properties.

Therefore, we asked whether exogenously added TH could affect stem cell properties in vitro. MuSCs freshly isolated by FACS and cultured in the presence of additional TH (3 nM T3 and T4) for 5 days showed reduced cell proliferation (Supplemental Figure 3, A and B). Interestingly, cells treated with TH remained in a Pax^+^EdU^–^ state compared with untreated controls, suggesting that TH treatment delayed the proliferation of MuSCs in vitro (Supplemental Figure 3, C and D).

### Genetic D2 depletion accelerates the early phase of muscle regeneration through a faster activation and proliferation of MuSCs.

**Figure 2:**
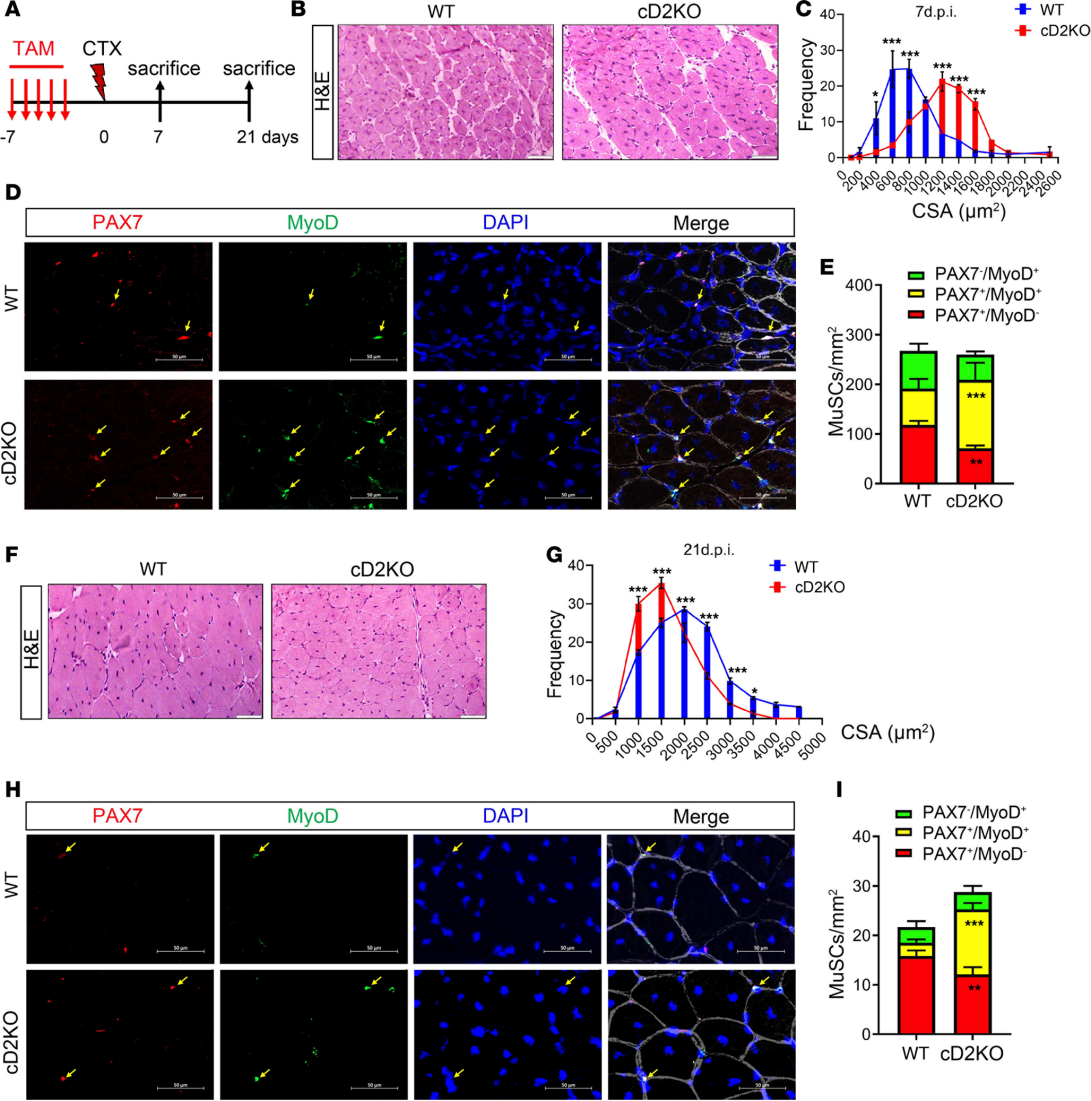
Absence of D2 leads to faster proliferation of MuSCs and transiently accelerates muscle regeneration. (**A**) Schematic of the experimental design, in which mice were euthanized at 7 (**B**–**E**) and 21 days (**F**–**I**). *n* = 9 WT and *n* = 9 cD2KO mice (7 days); *n* = 8 WT and *n* = 8 cD2KO mice (21 days). (**B**) H&E staining of the TA sections. Scale bars: 100 μm. (**C**) Quantification of the CSA in **B**. (**D**) Representative IF staining of PAX7 (red) and MyoD (green) on TA sections. The arrows indicate PAX7^+^MyoD^+^ cells. Scale bars: 50 μm. (**E**) The number of PAX7^–^MyoD^+^, PAX7^+^MyoD^+^, and PAX7^+^MyoD^–^ cells per mm^2^ in **D**. (**F**) H&E staining of the TA sections. Scale bars: 100 μm. (**G**) Quantification of the CSA. (**H**) Representative IF staining of PAX7 (red) and MyoD (green) on TA sections. The arrows indicate PAX7^+^MyoD^+^ cells. Scale bars: 50 μm. (**I**) The number of PAX7^–^MyoD^+^, PAX7^+^MyoD^+^, and PAX7^+^MyoD^–^ cells per mm^2^ in **H**. Data are presented as mean ± SEM; *P < 0.05, **P < 0.01, ***P < 0.001 using a Mann-Whitney test when comparing 2 conditions and 2-way ANOVA when comparing multiple conditions.

To evaluate the role of D2 in muscle regeneration, we analyzed TA muscles from cD2KO mice collected at 7 and 21 days after cardiotoxin (CTX) injection (Figure 2A). At 7 days after CTX injection, H&E staining and cross-sectional area (CSA) analysis revealed larger fiber sizes in cD2KO compared with WT mice (Figure 2, B and C). The number of activated Pax7^+^MyoD^+^ cells was increased upon D2 deletion compared with WT mice, whereas the number of quiescent Pax7^+^MyoD^–^ cells was reduced (Figure 2, D and E), indicating that D2 depletion promoted MuSC proliferation. This is not due to an increased number of satellite cells already present in D2KO mice at the time of injury (Supplemental Figure 4, A and B). Embryonic Myosin Heavy Chain (eMyHC) — a marker of regeneration — and laminin were already expressed as early as 3 days after injury in cD2KO, whereas they were barely detectable in WT mice (Supplemental Figure 4, C, Da, Db, and E). At 5 days after the injury, a residual increase in the expression of eMyHC was still observed in the cD2KO, whereas it rapidly increased in the WT at this time point (Supplemental Figure 4, C, Dc, Dd, and E). At 7 days, eMyHC staining was reduced in both mouse models (Supplemental Figure 4, C, De, Df, and E).

Later on, at 21 days after CTX injection, D2 depletion resulted in an increased number of fibers with a smaller diameter (Figure 2, F and G). While the number of Pax7^+^MyoD^+^ cells was still higher at this time in cD2KO mice compared with WT controls, the number of Pax7^+^MyoD^–^ cells was lower than that in WT (Figure 2, H and I). The faster activation of satellite cells led to an increased muscle mass in gastrocnemius measured at 21 days after injury (Supplemental Figure 5, A–C). FACS analysis of GFP^+^EdU^+^ cells at 21 days after injury confirmed the increased number of proliferating satellite cells (Supplemental Figure 5, D and E). Collectively, these data suggest that the absence of D2 promotes stem cell proliferation while delaying the completion of the myogenic program. This is consistent with the role of D2 in promoting cell differentiation at the late phase of myogenesis (19). Interestingly, 60 days after CTX injury, fiber diameter in cD2KO mice became larger than in that in WT, suggesting that the maturation delay in D2-depleted fibers can be overcome with prolonged time (Supplemental Figure 5, F–H), and this is in agreement with the larger skeletal muscle fibers that occur during hypothyroidism in humans (32).

Taken together, these data suggest that there is a more rapid activation of qMuSCs in cD2KO mice that allows a faster initiation of the regeneration. This advantage is lost later, when the completion of the myogenic program is delayed and does not occur normally in the absence of D2.

### D2 is required for self-renewal of qMuSCs.

A characteristic of stem cells is their ability to self-renew and to maintain a constant number. When proliferating MuSCs are induced to differentiate, a proportion of them escape terminal differentiation, arrest their cell cycle in the G_0_ phase, and return to quiescence. These mononuclear cells retain the self-renewal capacity, acquire stem cell properties, and become the so-called “reserve cells.” While the MyoD^+^ cells become positive for myogenin and irreversibly enter terminal differentiation, the other MyoD^–^ cells remain undifferentiated, resulting in the reserve cell population (33–36).

The reduction in the number of qMuSCs upon D2 depletion (Pax7^+^MyoD^–^; shown in Figure 2, D, E, H, and I) prompted us to investigate whether D2 plays a role in the return to quiescence, i.e., to analyze whether activated MuSCs can reenter quiescence in the absence of D2. To this end, freshly isolated MuSCs were cultured in growth medium for 3 days and induced to differentiate for 4 days after genetic removal of D2 with 4OH-TAM (Supplemental Figure 6A). We observed a reduced capacity to generate Pax7^+^MyoD^–^ cells (reserve cells) in the absence of D2, which was properly rescued by adding the D2 product T3 to the medium (Supplemental Figure 6, B and C). To ensure that we were properly assessing the self-renewing population, we also performed IF analysis for MyHC2 to mark differentiated cells and calculated the fusion index (Supplemental Figure 6, D and E). Notably, the fusion index was significantly lower in D2KO cells compared with WT and was also rescued in T3-treated cells (Supplemental Figure 6E). Collectively, these data indicate that D2KO MuSCs have impaired capacities for both self-renewal and to differentiate, both of which were rescued by the addition of T3. Consistent with this, while proliferating Pax7^+^MyoD^+^ cells were increased, Pax7^–^MyoD^+^ cells were slightly reduced upon D2 depletion (Supplemental Figure 6, B and C). Notably, a similar reduction in reserve cells was obtained by blocking D2 in MuSCs with rT3 treatment (Supplemental Figure 6, F and G).

**Figure 3:**
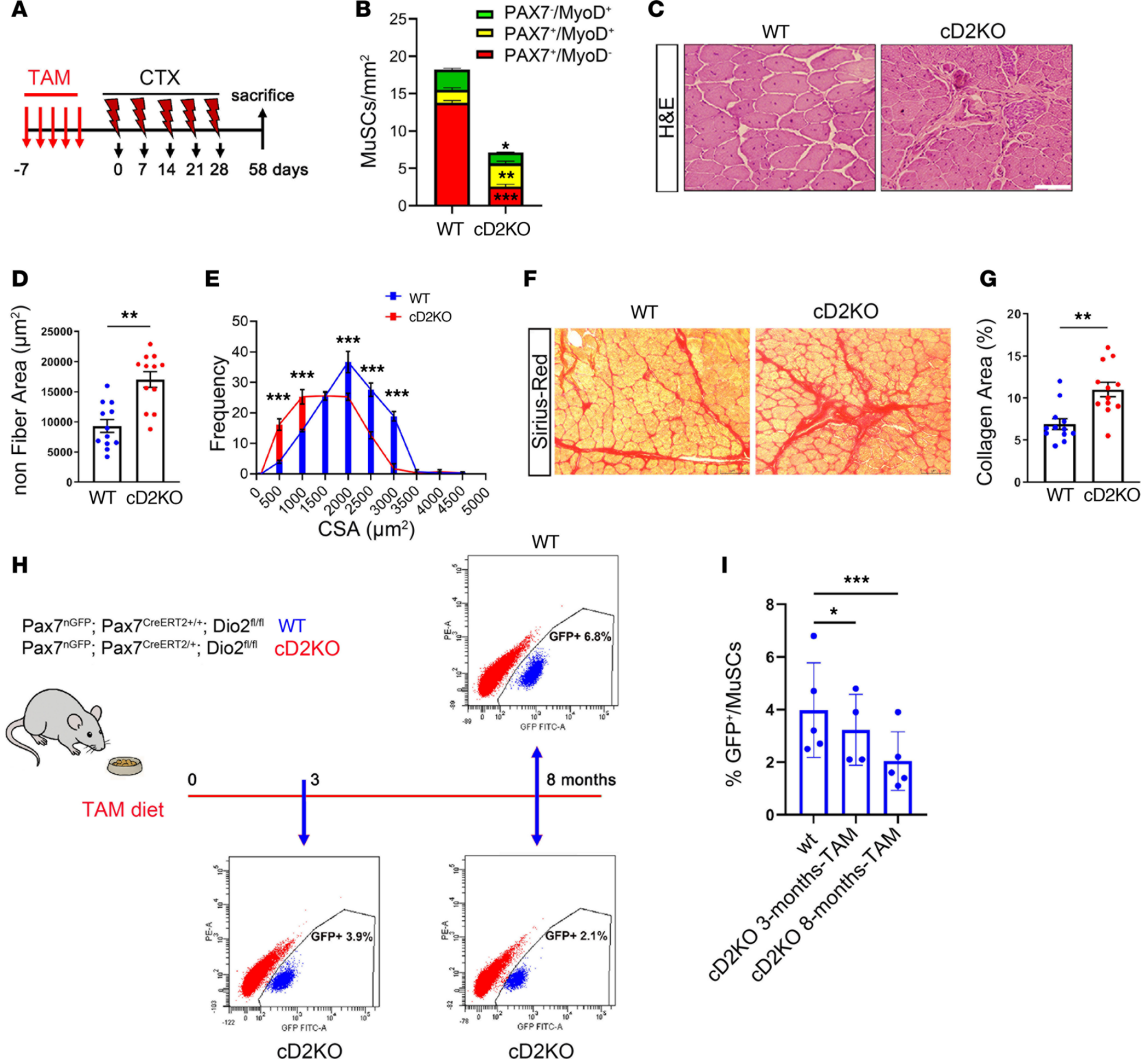
D2 is required for self-renewal of qMuSCs. (**A**) Schematic of the experiment with multiple CTX injections in cD2KO and WT mice. *n* = 12 WT and *n* = cD2KO mice. (**B**) The number of PAX7^–^MyoD^+^, PAX7^+^MyoD^+^, and PAX7^+^MyoD^–^ cells per mm^2^. (**C**) H&E of TA sections. Scale bar: 75 μm. (**D**) Percentage of nonmyofiber tissue. (**E**) Quantification of the CSA of TA sections. (**F**) Representative Sirius Red staining of TA sections. Scale bars: 75 μm. (**G**) Percentage of collagen area in **F**. (**H**) Mouse model used and diagram of the experimental design. FACS analysis showing GFP^+^ /MuSC cells at 3 and 8 months after D2 depletion compared with WT mice. *n* = 5 WT, and *n* = 4 (3 months) and *n* = 8 (8 months) cD2KO mice. (**I**) Quantification of GFP^+^/MuSCs in **H**. Data are presented as mean ± SEM; *P < 0.05, **P < 0.01, ***P < 0.001 using a Mann-Whitney test when comparing 2 conditions and 2-way ANOVA when comparing multiple conditions.

To evaluate whether D2-depleted MuSCs could normally reconstitute the stem cell pool in vivo, we analyzed muscle repair after 5 consecutive CTX injuries, at 30 days after the last CTX (Figure 3A). After multiple injuries, the number of quiescent (Pax7^+^MyoD^–^) MuSCs was lower in cD2KO mice (Figure 3B). Furthermore, the areas of non-muscle tissue were larger in cD2KO mice compared with control (Figure 3, C and D), while the diameter of the formed muscle fibers was reduced (Figure 3E). Correspondingly, we observed increased intramuscular fibrosis, confirming the partial inability of MuSCs to regenerate in cD2KO after successive injuries (Figure 3, F and G). Collectively, these data indicate a self-renewal deficiency of D2-depleted MuSCs, leading to a gradual depletion of resident stem cells and a consequent increased fibrosis after multiple muscle injuries.

To measure the effects of long-term D2 depletion in resting muscles, we quantified the number of satellite cells by FACS analysis at both 3 and 8 months after D2 depletion. Importantly, a significant reduction in the number of D2-depleted MuSCs was observed compared with WT cells (Figure 3, H and I).

To further expand on the long-term consequences of D2 depletion in resting conditions, we compared Tg:Pax7^CreERT2/+^ R26^mTmG^ D2^fl/fl^ with Tg:Pax7^CreERT2/+^ R26^mTmG^ D2^+/+^ 4 months after D2 depletion (Supplemental Figure 6H). Under resting conditions, we observed that the newly generated fibers receiving D2-depleted MuSCs (green in Supplemental Figure 6I) were more numerous and smaller compared with WT fibers (Supplemental Figure 6, I–K).

Taken together, these results suggest that in the long-term, physiological stimuli of muscle turnover, when acted upon abnormally alerted MuSCs as in chronic D2 depletion, determine an increased number of newly generated fibers compared with WT muscles and a reduction in the stem cell pool.

### D2 sustains the Notch pathway in quiescent cells.

**Figure 4:**
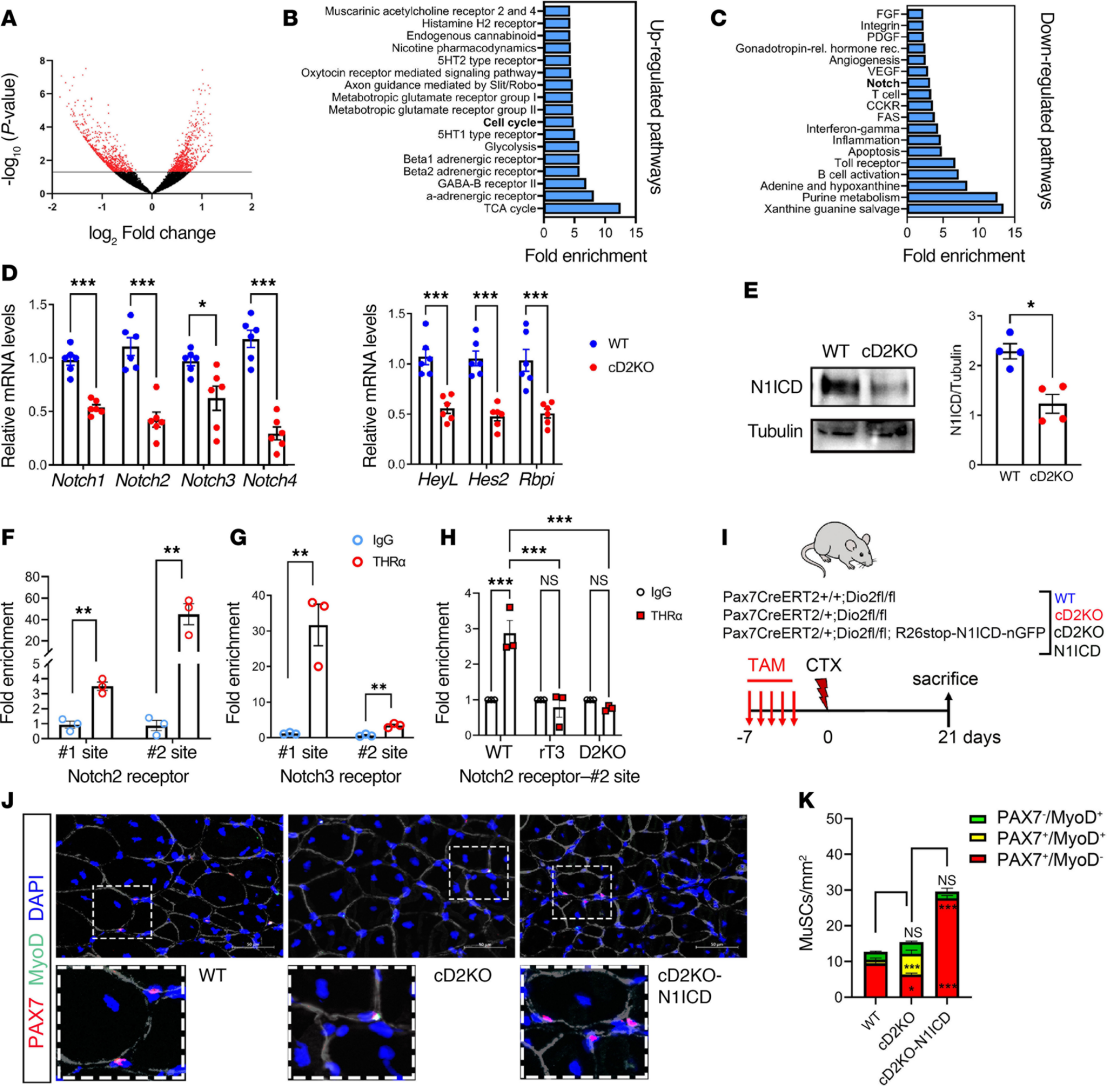
D2 action sustains the Notch signaling pathway. (**A**) Volcano plot showing differences in the mRNA expression of qMuSCs from cD2KO and WT mice. Negative log_10_ *P* value (*y* axis) and log_2_ fold change (*x* axis) are plotted for transcripts detected by RNA-seq analysis. The experiments were conducted on *n* = 3 biological replicates. Gray line indicates *P* value < 0.05. (**B**) The top upregulated pathways in Panther (Protein Analysis Through Evolutionary Relationships; http://www.pantherdb.org/). (**C**) The downregulated pathways in Panther. (**D**) mRNA levels of Notch receptors (Notch1–4) and Notch targets (HeyL, Hes2, and Rbpi) in freshly isolated qMuSCs by FACS. *n* = 6 WT and *n* = 6 cD2KO mice. (**E**) Western blot of cD2KO and WT qMuSCs freshly isolated by FACS. The graph on the right shows the quantification of N1ICD/tubulin. (**F** and **G**) ChIP-qPCR using THR-α and isotype IgG control antibodies on proximal enhancer and promoter regions of Notch2 and Notch3 genes, respectively, in C2C12 cells. Data are normalized to input chromatin; *n* = 3 biologically independent samples. (**H**) ChIP-qPCR using THR-α and isotype IgG control antibodies on proximal enhancer of Notch2 in freshly isolated MuSCs from WT, rT3-treated, and D2KO cells. Data are normalized to input chromatin; *n* = 3 biologically independent samples. (**I**) Mouse model used and diagram of in vivo rescue experiment. 2 independent experiments were conducted with *n* = 4 WT, *n* = 4 cD2KO, and *n* = 4 cD2KO-N1ICD mice each. (**J**) Representative IF of PAX7 (red) and MyoD (green) in TA muscle from indicated mice harvested 21 days after CTX injection. Scale bars: 50 μm. (**K**) The number of PAX7^–^MyoD^+^, PAX7^+^MyoD^+^, and PAX7^+^MyoD^–^ cells per mm^2^. Bars represent the average of at least 3 technical replicates. *n* = 8 mice for each group. Data are presented as mean ± SEM; *P < 0.05, **P < 0.01, ***P < 0.001 using a 2-tailed Student’s *t* test when comparing 2 conditions and 2-way ANOVA when comparing multiple conditions.

We analyzed the cellular transcriptome in qMuSCs isolated from resting muscles of cD2KO versus WT mice. Approximately 1,300 genes were significantly altered by D2 depletion, of which approximately half were upregulated, and half were downregulated (Figure 4A). By functional analysis, we identified several pathways affected by D2 depletion. Interestingly, among the upregulated pathways, cell cycle stood out as a significant pathway induced by D2 depletion (Figure 4B), whereas among the downregulated pathways, Notch signaling stood out as a potential regulator of stemness (Figure 4C).

The expression of key components of the Notch pathway was significantly reduced in FACS-isolated qMuSCs from cD2KO mice, including Notch target genes (Heyl, Hes2, and Rbpj) and Notch receptors (Notch1–4) (Figure 4D). Consistently, protein levels of the active Notch1 intracellular domain (N1ICD) were reduced in D2-depleted qMuSCs (Figure 4E). TH treatment for 24 and 60 hours induced a time-dependent increase in N1ICD levels, indicating progressive activation of Notch signaling, and was accompanied by increased expression of downstream Notch target genes (Supplemental Figure 7, A–D).

To determine whether TH regulates Notch target gene expression exclusively through canonical Notch activation, cells were treated with the γ-secretase inhibitor DAPT in the presence or absence of TH. DAPT effectively suppressed N1ICD generation (Supplemental Figure 7E). Despite blockade of Notch activation, TH maintained induction of Heyl and Notch3 expression, whereas Hes1 and Hes2 expression was markedly reduced (Supplemental Figure 7, F–I), indicating differential dependence of Notch-associated genes on N1ICD-mediated signaling.

We next assessed direct binding of TH receptor alpha (THRα) to regulatory regions of Notch2 and Notch3. ChIP analysis demonstrated robust THRα occupancy at promoter/proximal regions of both loci (Supplemental Figure 7J), with up to approximately 40-fold enrichment at Notch2 and approximately 35-fold enrichment at Notch3 (Figure 4, F and G). Notably, THRα binding at these sites was reduced to background levels in rT3-treated or D2KO cells (Figure 4H and Supplemental Figure 7, K–M).

Together, these results demonstrate that D2-dependent TH signaling promotes Notch pathway activity in qMuSCs through both canonical Notch activation and direct THRα-mediated transcriptional regulation of some Notch-related genes, including Notch2 and Notch3.

To assess whether active Notch could functionally rescue D2 depletion in vivo, we generated Pax7^CreERT2/+^ D2^fl/fl^ R26^stop-N1ICD-nGFP^ (hereafter referred to as cD2KO-N1ICD) mice in which D2 was deleted in MuSCs with simultaneous Notch overactivation (Figure 4I). We compared the ability of the MuSC population from cD2KO-N1ICD and cD2KO mice to regenerate TA muscles 21 days after CTX injury. IF staining revealed a significant reduction in the number of activated Pax7^+^MyoD^+^ cells and a corresponding increase in quiescent Pax7^+^MyoD^–^ cells in cD2KO-N1ICD mice compared with cD2KO control (Figure 4, J and K). These data indicate that D2 is required for full, but not absolute, activity of the Notch pathway in qMuSCs and that forced Notch activity can rescue some of the effects of D2 depletion.

### Acute D2 depletion in resting muscle turns qMuSCs into a G_Alert_-like state.

**Figure 5:**
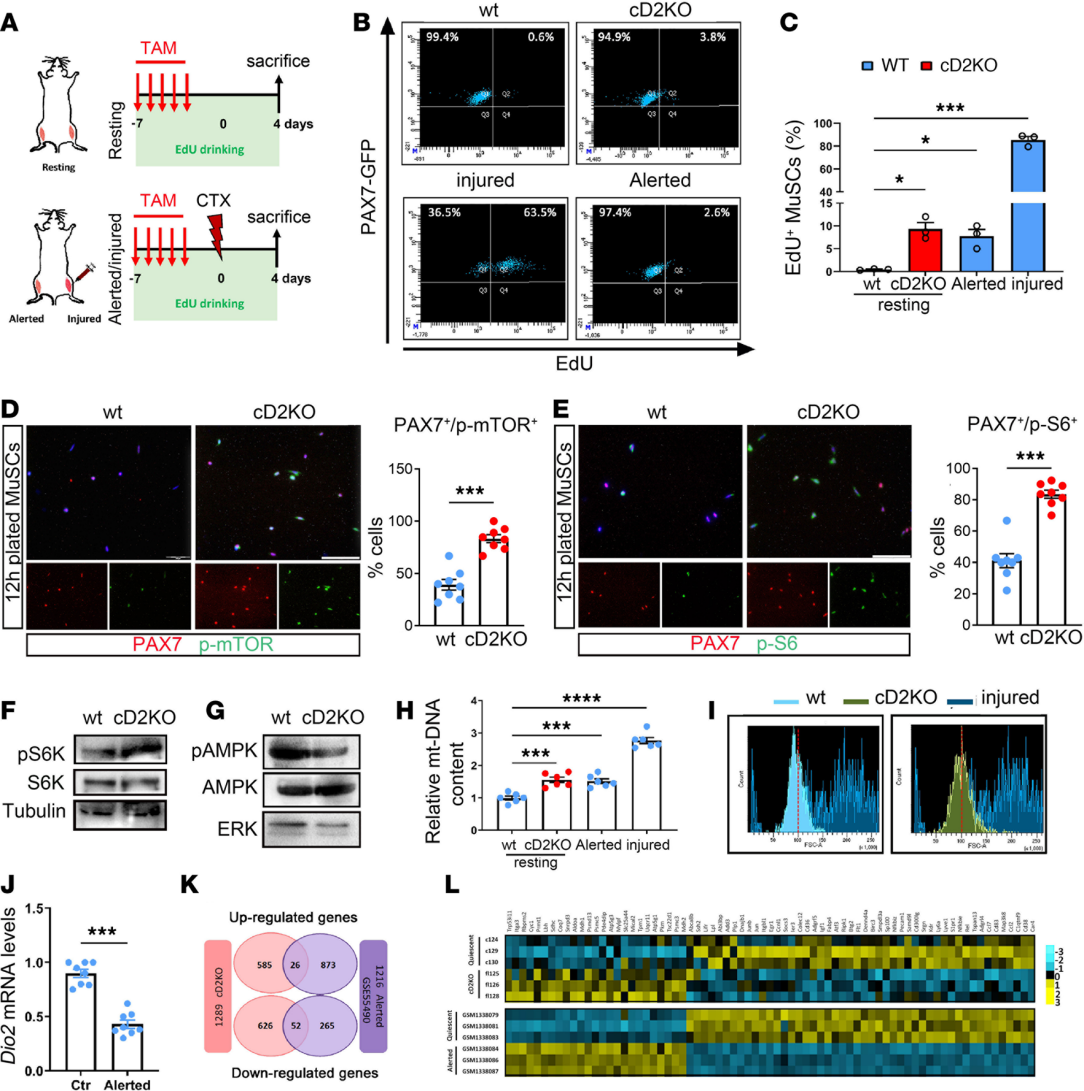
D2 depletion turns qMuSCs to an alert state. (**A**) Schematic of the experiment. (**B**) Representative FACS plots for EdU/Pax7-GFP of MuSCs isolated from resting TA muscle of indicated mice (upper panels) and from alerted and injured muscles of WT mice (lower panels). (**C**) Percentage of EdU^+^/MuSCs in **B**. *n* = 4 independent experiments with *n* = 3 WT and cD2KO mice. (**D** and **E**) Representative IF of PAX7 and p-mTOR (**D**) and p-S6 (**E**) in FACS-isolated MuSCs from resting WT and cD2KO mice (scale bars: 200 μm) and respective quantification of the percentage of PAX7^+^p-mTOR^+^ cells (**D**) and PAX7^+^p-S6^+^ cells (**E**). *n* = 8 WT and *n* = 8 cD2KO mice. (**F** and **G**) Western blots of p-S6K and total S6K (**F**) and p-AMPK and total AMPK (**G**). Tubulin and ERK served as a loading control. (**H**) Relative content of mtDNA in FACS-isolated MuSCs from resting WT, resting cD2KO, alerted WT, and injured WT mice muscles. *n* = 6 WT and cD2KO mice. (**I**) Representative FACS histogram of forward scatter (FSC) signal of MuSCs from resting WT (light blue), resting cD2KO (green), and injured WT (blue) muscles. (**J**) Dio2 mRNA levels of MuSCs from WT muscles in resting (Ctr) and alerted conditions. Alerted MuSCs were harvested 2 days after CTX in contralateral TA muscle. *n* = 8 WT and cD2KO mice. (**K**) Venn comparison analysis between cD2KO RNA-seq and G_Alert_ state array (10); common genes downregulated (*P* < 2.418 × 10^–16^) and common genes upregulated (*P* < 0.041) are shown. (**L**) Heatmap comparison of common genes up/downregulated from cD2KO RNA-seq versus control (quiescent) and G_Alert_ state array previously described (10). Data are presented as mean ± SEM; *P < 0.05, **P < 0.01, ***P < 0.001 using a Mann-Whitney test when comparing 2 conditions, and 2-way ANOVA when comparing multiple conditions.

As mentioned, cell cycle was among the upregulated pathways induced by D2 depletion in MuSCs isolated from resting muscles (Figure 4B). Therefore, we compared by FACS analysis the percentage of EdU^+^ MuSCs in D2-depleted versus WT muscles in resting, injury, and alerted conditions (Figure 5A). We observed that although the percentage of EdU^+^ in D2-depleted MuSCs did not reach that of injured conditions, it approached that of alerted MuSCs in WT muscles (Figure 5, B and C).

qMuSCs transition between a dormant G_0_ state and a primed, nondividing G_Alert_ state induced by systemic injury (10). To determine whether D2 depletion alone is sufficient to induce a G_Alert_ state in MuSCs, we analyzed established G_Alert_ markers in MuSCs freshly isolated by FACS from resting muscles of cD2KO and WT mice. D2-depleted MuSCs displayed activation of mTORC1 signaling, as shown by increased p-mTOR (Figure 5D), p-S6 (Figure 5E), and p-S6K (Figure 5F), concomitant with reduced p-AMPK levels (Figure 5G). Consistent with a G_Alert_ phenotype, D2-depleted MuSCs showed increased mtDNA content (Figure 5H) and larger cell size (Figure 5I). These data indicate that D2 depletion is sufficient to induce a G_Alert_ state in qMuSCs in vivo.

In parallel, we also examined whether a change in D2 levels occurs in alerted WT qMuSCs. Interestingly, D2 expression was reduced in G_Alert_ cells compared with quiescent G_0_ cells (controls) (Figure 5J), consistent with the observation that D2 depletion triggers the alerting process. Lastly, we compared in silico the genes differentially expressed in cD2KO versus WT MuSCs with those induced in the G_Alert_ state deposited in the Gene Expression Omnibus (GEO) database (GSE55490; Figure 5K) (10). Interestingly, of the 678 downregulated genes in cD2KO qMuSCs, 52 overlap with the downregulated genes in the G_Alert_ state (*P* < 2.418 × 10^–16^), whereas of the 611 upregulated genes in cD2KO qMuSCs, 26 overlap with the upregulated genes in the G_Alert_ state (*P* < 0.041) (Figure 5L). Collectively, these data indicate that D2 depletion in qMuSCs induces a state in which the transcriptional signature significantly overlaps with the G_Alert_ state, supporting the finding that D2 depletion alone is sufficient for the transition of qMuSCs from the G_0_ to the G_Alert_-like state.

### Characterization of the mdx-D2KO mice and transplantation assays to evaluate cell engrafting potential in vivo of D2-depleted cells.

**Figure 6:**
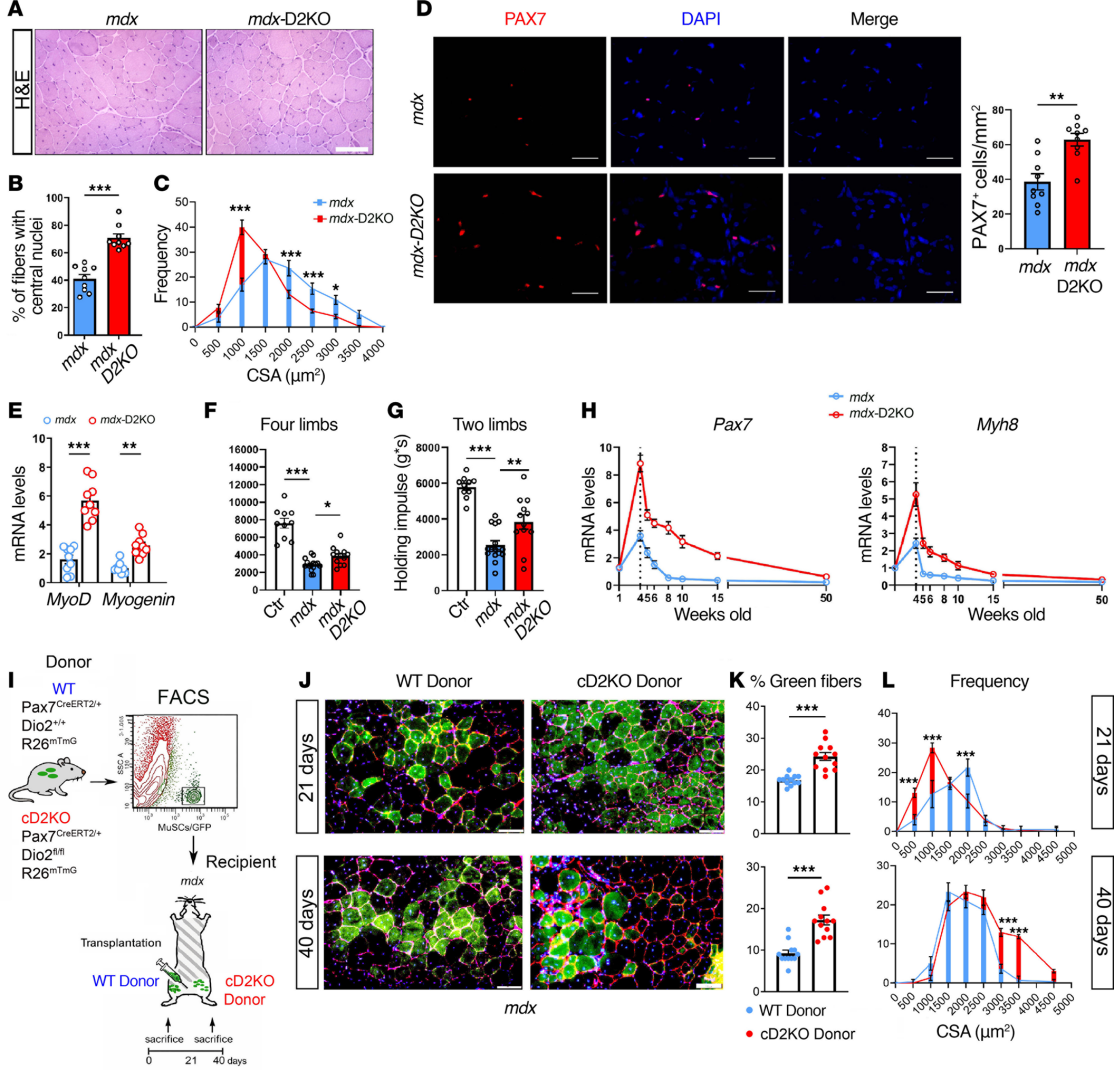
D2KO improves the phenotype of mdx mice. (**A**) Representative H&E of TA sections from mdx and mdx-D2KO mice. Scale bar: 100 μm. *n* = 9 mdx and mdx-D2KO mice. (**B**) Quantification of the percentage of fibers with central nuclei in mdx and mdx-D2KO mice. (**C**) Quantification of the CSA. (**D**) Representative IF staining of PAX7^+^ (scale bars: 100 μm) and quantification (right). (**E**) Relative mRNA levels of MyoD and myogenin. (**F** and **G**) Performance in 4-limb (**F**) and 2-limb (**G**) muscle strength hanging tests of controls (Ctr), mdx, and mdx-D2KO mice at 4 weeks of age. *n* = 10 Ctr, *n* = 15 mdx, and *n* = 12 mdx-D2KO mice. (**H**) Pax7 and Myh8 mRNA levels from muscles at different weeks of age. Data are presented as mean ± SEM of at least 3 technical replicates. *n* = 6 mdx and *n* = 6 mdx-D2KO mice. (**I**) Schematic of the D2-depleted MuSC transplantation assay. MuSC/GFP^+^ cells from WT (Pax7^creERT2/+^ D2^+/+^ R26^mTmG^) and cD2KO (Pax7^creERT2/+^ D2^fl/fl^ R26^mTmG^) mice were transplanted into the TA muscle of a single recipient mdx mouse. *n* = 12 WT and *n* = 12 cD2KO recipient mdx mice. (**J**) Representative IF staining of green epifluorescent fibers of mdx mice xenografted as indicated. Laminin is in red. Scale bars: 100 μm. (**K**) Percentage of green epifluorescent fibers at 21 (upper) and 40 days (lower) following transplantation of MuSC/GFP cells from WT and cD2KO donors. (**L**) Quantification of the CSA (μm^2^) of green fibers 21 (upper) and 40 days (lower) after xenografting. Data are presented as mean ± SEM of at least 3 technical replicates; *P < 0.05, **P < 0.01, ***P < 0.001 using a Mann-Whitney test when comparing 2 conditions and 2-way ANOVA when comparing multiple conditions. Each point (in **B**, **D**–**G**, and **K**) represents the average of at least 3 technical replicates from each mouse.

Premature exhaustion of MuSCs is a major determinant of the dystrophic process (37, 38). Therefore, we asked whether D2 depletion could affect the dystrophic phenotype of *mdx* mice. To this end, we generated *mdx*/global D2 knockout (*mdx*-D2KO) mice and characterized the muscle phenotype using morphological, molecular, and functional analyses. At postnatal week 4, which corresponds to the peak of dystrophy, fiber size was smaller and the number of fibers with central nuclei was increased in *mdx*-D2KO mice compared with *mdx* mice (Figure 6, A–C). An increased number of Pax7^+^ cells was detected in the TA muscle of *mdx*-D2KO mice compared with *mdx* mice (Figure 6D). In addition, myogenin and MyoD mRNA levels were higher in *mdx*-D2KO mice compared with *mdx* controls (Figure 6E).

To assess whether D2 depletion functionally improves muscle performance in dystrophic mice in vivo, we tested *mdx*-D2KO versus *mdx* mice with both 2- and 4-limb hanging tests (in each case represented as the holding impulse). We observed that *mdx*-D2KO mice exhibited a significantly increased ability to resist their gravitational force compared with *mdx* mice (Figure 6, F and G). Finally, we measured Pax7 and neonatal MHC mRNA levels in a period from postnatal weeks 4 to 50 and found that both markers were higher in *mdx*-D2KO mice than in *mdx*^–^ controls (Figure 6H). Overall, these results show that global D2 deletion in the *mdx* context results in a markedly improved muscle dystrophic phenotype.

Next, to assess whether D2 depletion translates into a cell-autonomous regenerative advantage, we tested the functional capacity of D2-depleted MuSCs to engraft in vivo by performing a transplantation assay. We isolated MuSCs from donor Tg:Pax7^CreERT2/+^ R26^mTmG^ D2^fl/fl^ versus Tg:Pax7^CreERT2/+^ R26^mTmG^ D2^+/+^ mice and injected them into the TA of *mdx* mice (Figure 6I). The engraftment of donor-derived MuSCs into *mdx* recipients was analyzed by direct epifluorescence for GFP on TA muscle harvested 21 and 40 days after transplantation (Figure 6J). The percentage of GFP^+^ D2-depleted fibers was increased at both 21 and 40 days after transplantation (Figure 6K), consistent with the increased proliferative capacity of D2-depleted cells. CSA analysis showed that D2-depleted fibers were smaller at 21 days and larger at 40 days compared with the control, indicating that the D2-dependent maturation defect could be compensated at a later time (Figure 6L), which is consistent with what was observed in cD2KO mice after injury (Figure 2G versus Supplemental Figure 5H).

Overall, these results indicate that D2-depleted MuSCs successfully engraft and have an increased proliferative capacity and a time-dependent delay in maturation that is compensated at later time points.

### D2 depletion promotes the activation of stem cells in the skin.

To assess whether D2 expression in stem cells is restricted to muscle or is a common feature in other tissues, we analyzed the localization of D2 in skin. Interestingly, D2 expression colocalized with the hair follicle stem cell marker CD34, which marks the bulge region of the hair follicle where quiescent stem cells are localized (Supplemental Figure 8A). In addition, we found that D2 expression is dynamically regulated during the hair follicle cycle. Indeed, D2 was clearly detectable in telogen, the quiescent phase of the hair follicle cycle, almost absent in anagen and catagen, and never coincided with EdU labeling (Supplemental Figure 8B). scRNA-seq analysis of full-thickness mouse skin (39) revealed that D2 is expressed predominantly in the epidermis and outer bulge region and directly correlates with the expression of putative skin stem cell markers in these regions (e.g., CD34 and Krt15), whereas it is not expressed in activated hair germ cells. Consistent with its expression primarily in quiescent stem cells, D2 expression is reduced in the anagen phase of the hair follicle (Supplemental Figure 8C).

To investigate the functional role of D2 in the hair follicle stem cells, we generated an inducible skin conditional D2KO (scD2KO) mouse model (Tg:K14^CreERT2/+^ D2^fl/fl^). Interestingly, the percentage of CD34^+^/integrin-α6^+^ cells (i.e., hair follicle stem cells) was significantly increased 6 days after D2 depletion (Supplemental Figure 8D), suggesting that, similar to what we observed in muscle, blocking D2 in hair follicle stem cells promotes their activation.

Next, we analyzed the effects of specific D2 depletion in the hair follicle compartment of mice subjected to 1 (T1) or 3 (T3) consecutive rounds of hair depilation. While D2 depletion increased the number of putative stem cells (Sox9^+^EdU^+^) in the hair follicle bulges after a single round of depilation (T1), we observed a net decrease of Sox9^+^EdU^+^ cells at T3 compared with that in WT mice (Supplemental Figure 8E). Correspondingly, *Sox9* and *CD34* mRNAs were initially increased at T1, while their expression was reduced at T3 in scD2KO compared with WT controls (Supplemental Figure 8F). This suggests that while D2 depletion initially increased the activation of skin stem cells, it led to stem cell exhaustion after subsequent rounds of amplification.

To characterize the effects of D2 depletion in vivo during skin regeneration, we performed a wound healing assay comparing scD2KO mice to controls. A time-course experiment of regenerating epidermis showed that wound closure was significantly faster in D2-depleted epidermis than in control skin (Supplemental Figure 8G). Overall, these data indicate that D2 plays an important role in hair follicle stem cells and that, importantly, its depletion enhances wound repair in adult mouse skin.

### Transient pharmacological D2 inhibition accelerates tissue repair in injured muscle and skin.

To overcome the limitations of the genetic D2KO model, namely, its irreversibly depletion, we used rT3 to suppress D2 activity only within a defined temporal window. First, we assessed whether MuSCs isolated from rT3-treated mice behaved similarly to those from D2KO mice under resting conditions. We found that the active intracellular domain of Notch (N1ICD) was reduced in MuSCs isolated from rT3-treated mice compared with controls (Supplemental Figure 9, A and B). Moreover, these cells exhibited a G_Alert_-like state, as demonstrated by the EdU^+^MuSC^+^ FACS analysis (Supplemental Figure 9, C–E), both consistent with the phenotype of D2KO mice (Figure 4E and Figure 5C).

**Figure 7:**
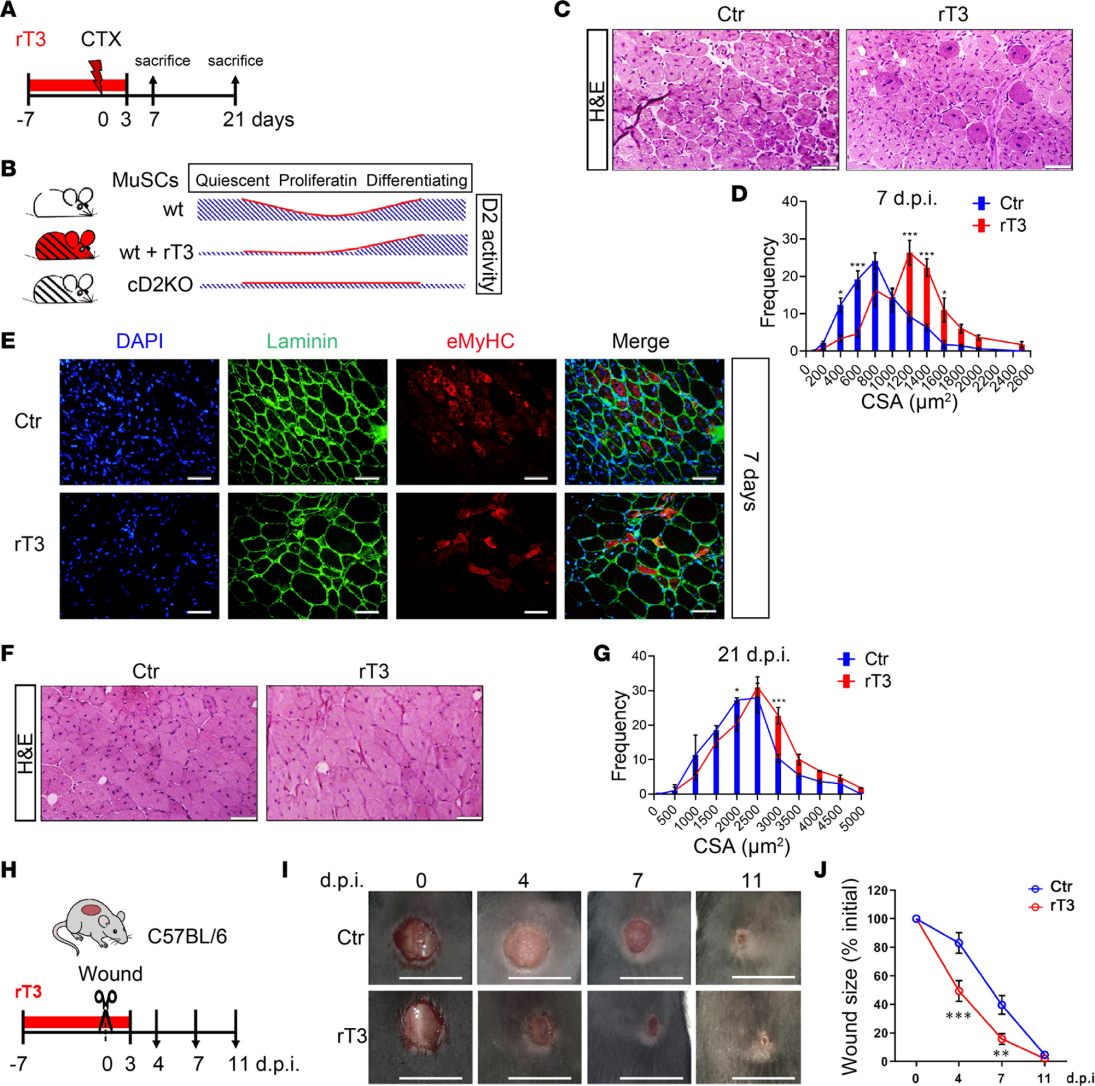
Pharmacological D2 blockade as an agent to enhance repair of muscle and skin injury. (**A**) Schematic illustrating the experimental design. 2 independent experiments were conducted (*n* = 8 untreated and *n* = 10 rT3-treated mice, each). In each experiment, *n* = 4 untreated and *n* = 5 rT3-treated mice were euthanized at 7 and 21 days after CTX injury. (**B**) Schematic of D2 activity in MuSCs during myogenesis in untreated WT, rT3-treated WT, and untreated cD2KO mice. (**C**) Representative H&E staining of TA sections (scale bars: 50 μm) from untreated (Ctr) and rT3-treated mice euthanized 7 days after CTX injury. (**D**) Quantification of the CSA of untreated and rT3-treated mice euthanized 7 days after CTX injury. (**E**) Representative IF staining for eMyHC (red) and laminin (green) in untreated and rT3-treated mice euthanized 7 days after CTX injury. Scale bars: 50 μm. (**F**) Representative H&E staining of TA sections (scale bars: 50 μm) of untreated and rT3-treated mice euthanized 21 days after CTX injury. (**G**) Quantification of the CSA of untreated and rT3-treated mice euthanized 21 days after CTX injury. (**H**) Diagram of the experimental design of wound healing in untreated and rT3-treated mice. *n* = 12 WT mice and *n* = 12 rT3-treated mice. (**I**) Representative images of the wound healing experiment at wound creation (0) and after 4, 7, and 11 days. Scale bars: 1 cm. (**J**) Quantification of wound closure expressed as the mean percentage decrease compared with the initial wound size. Data are presented as mean ± SEM; *P < 0.05, **P < 0.01, ***P < 0.001 using 2-way ANOVA.

We sought to determine whether pharmacological D2 blockade in a specific time window could enhance tissue recovery after injury in muscle and skin. We used rT3 to specifically block D2 activity only at the early phase of regeneration, while allowing proper D2 action at later time points (Figure 7, A and B). We treated mice with oral rT3 for 10 days (–7/+3 relative to CTX injury) and euthanized them at 7 and 21 days after CTX injury (Figure 7A). Analysis at 7 days after CTX injury revealed larger fibers in the muscles of rT3-treated mice compared with untreated WT controls (Figure 7, C and D), similar to what was observed in the cD2KO mice. Also similar to cD2KO mice (as shown in Supplemental Figure 4, C–E), eMyHC expression was reduced in muscles from rT3-treated mice 7 days after CTX injury compared with untreated WT controls (Figure 7E). However, differently from cD2KO mice, the analysis at 21 days after CTX injury showed larger fibers in rT3-treated mice compared with untreated WT controls (Figure 7, F and G), indicating that the maturation delay caused by the absence of D2 did not occur upon transient rT3 treatment. This phenotype is consistent with an enhancement of stem cell activation by the absence of D2 during the early stage of MuSC activation and with a functioning D2 during the later stage of maturation that prevents the delay in fiber maturation observed in the cD2KO mice. Importantly, these data suggest that a transient pharmacological blockade of D2 in injured muscle increases MuSC activation by prompting qMuSCs in a G_Alert_-like state, thus accelerating muscle regeneration without negatively affecting the differentiation phase.

In a similar setting, we tested whether pharmacological D2 inhibition positively affects skin wound healing. Concordantly, we found that rT3-treated mice, either before the wound or contemporaneously, repaired wounds faster than control mice (Figure 7, H–J, and Supplemental Figure 9, F and G).

In conclusion, we have shown that drug-induced D2 blockade within a specific time frame facilitates regeneration in different tissues and could be therapeutically exploited in vivo.

## Discussion

Cellular quiescence is a property of several adult vertebrate stem cells. How this state is regulated and maintained is a central question in stem cell biology. Here, we report that the TH-producing enzyme D2 is a key metabolic player that acts as a regulator of MuSC quiescence. The D2-mediated action represents a functional link between the circulating TH concentrations and a cell-autonomous mechanism that allows to customize/increase intracellular TH to preserve quiescence and stem cell function.

The temporal expression pattern of D2 in MuSCs is peculiar. Indeed, *Dio2* mRNA is highly expressed in quiescence, drastically reduced upon activation, and turned on again at a later stage of myogenesis to allow full differentiation (Supplemental Figure 1B). The described pattern of D2 expression is consistent with what was previously reported in mRNA datasets from quiescent and activated MuSCs (GEO GSE47177) (40) (Supplemental Figure 1M). Our data suggest that D2 is reexpressed also in a small subset of activated MuSCs and that the D2 reexpression is relevant to their return to quiescence (Supplemental Figure 6, A–C). In the absence of D2, proliferating myoblasts fail to renew quiescent cells, ultimately leading to depletion of the stem cell pool (Figure 3, H and I, and Supplemental 6, A–C).

The role of D2 in quiescence was previously unknown. Given the differentiating and antiproliferative effects of TH in multiple contexts (19, 41), we propose that elevated intracellular TH in muscle and skin stem cells helps maintain quiescence by buffering activating cues from the niche. Upon niche perturbation, rapid D2 downregulation facilitates exit from quiescence. The signals that regulate D2 expression in quiescent cells are currently unknown and represent the aim of future studies. The identification of D2 as specifically expressed in quiescent stem cells adds TH signaling to the list of key players in the mechanisms controlling quiescence. Importantly, this study highlights the importance of the D2-produced T3 at the cellular level in the stem cell compartment. The data from cD2KO mice indicate that the normal circulating T3 concentration in the bloodstream is not sufficient and that an additional amount of D2-produced T3 is required to preserve quiescence.

What are the molecular mechanisms by which D2 controls quiescence? RNA-seq analysis from qMuSCs revealed that multiple pathways/signals are altered upon D2 genetic depletion, including Notch (Figure 4, B and C). The Notch pathway is a key regulator of many types of adult stem cells in a variety of tissues (29). qMuSCs are characterized by elevated Notch activity, which is required for the maintenance of undifferentiated state (42, 43).

Here, we shown that the THRα directly binds to the Notch2 and Notch3 gene loci and promotes their expression (Figure 4, F and G, and Supplemental Figure 7J). Importantly, D2 depletion — whether induced pharmacologically or genetically — results in a marked reduction in THRα occupancy at the Notch2 and Notch3 regulatory regions (Figure 4H and Supplemental Figure 7, K–M). These findings demonstrate that local D2 activity is required to sustain THR binding at these loci and directly influences THR binding at DNA binding sites. The functional association between Notch and D2 was initially suggested by the expression profile of Pax7-^nGFP^ sorted cells, where both D2 and Notch expression reached their highest levels in the GFP^hi^ MuSC subpopulation (Supplemental Figure 1A) (18). Using different combinations of genetically modified mice with altered D2 and Notch levels, we found that D2 is epistatically located upstream of Notch in qMuSCs. Notch overexpression could partially rescue the phenotype observed upon D2 depletion in vivo (Figure 4, I–K). However, given the pleiotropic effects of TH, it is likely that other genes/pathways regulated by TH and not compensated by Notch may influence the MuSC behavior. Of note, differences in Notch signaling could also reflect intrinsic heterogeneity among MuSC subpopulations (e.g., MyoD^+^ versus MyoD^–^ cells) rather than exclusively direct transcriptional regulation of Notch genes by THR within an otherwise equivalent cell population.

Reserve experiments also indicated that D2 is required to return proliferating progenitor myoblasts to quiescence (Figure 3I and Supplemental Figure 6, A–E). In vivo, D2 depletion resulted in a functional deficiency to restore the stem cell reservoir in the event of multiple challenges, as observed in muscle (Figure 3, A and B) and skin (Supplemental Figure 8, E and F). The role of Notch signaling in MuSC self-renewal is consistent with the reduced self-renewal capacity observed in cD2KO cells. In this scenario, the impairment in self-renewal in D2KO MuSCs could be consequent to the downregulation of Notch. Accordingly, the decline in Pax7^+^MyoD^–^ cells in cD2KO mice was rescued by N1ICD overactivation. Given the distinct roles of Notch and TH, the phenotype resulting from D2 depletion only partially overlaps with that of Notch impairment in vivo (43). Moreover, stem cell number is tightly tuned to an optimal physiological set point, and attempts to artificially enhance it are likely to have long-term detrimental effects.

What are the consequences of D2 depletion in qMuSCs? We found that specific D2 depletion in resting muscles induces the transition from G_0_ to a G_Alert_-like state, while in the regenerative setting, it promotes cell proliferation. The transition from G_0_ to G_Alert_ was first demonstrated in resting cells by the action of systemic HGFA released upon muscle injury (12). Here, we found that a decrease in intracellular TH concentration by D2 depletion is sufficient to induce a G_Alert_-like state in the absence of other systemic cues. Mechanistically, we found that D2 depletion activates mTOR and S6K and causes a corresponding reduction in p-AMPK (Figure 5, D–G). The effects of D2 depletion and the corresponding reduction in TH concentration are consistent with the previously reported effects of TH treatment on stimulating p-AMPK levels in C2C12 myoblasts (44). In this scenario, it is reasonable to hypothesize that the known ability of TH to induce rapid mobilization of intracellular Ca^2+^ levels (44, 45) could act as a molecular trigger for calcium/calmodulin-dependent protein kinase kinase 2 activity and the subsequent p-AMPK/mTORC signaling cascade. Interestingly, the molecular gene program triggered by D2 depletion is significantly consistent with the gene signature triggered by the alerted state (Figure 5, K and L), strongly supporting the notion that there is a large overlap between D2 depletion and the transition to G_Alert_. Importantly, the mechanisms that cause entry into G_Alert_ also determine a reduction in D2 levels in vivo. The reduced level of D2 expression measured in alerted MuSCs (Figure 5J) provides important support for the association between G_Alert_ and D2 action. The molecular mechanisms for this are currently unknown, but it is reasonable to hypothesize an inhibition of D2 expression by circulating growth factors (e.g., HGFA) that normally act as alarmins in the plasma.

The phenotype observed upon D2 deletion is different from what was observed upon Notch deletion. Notably, while in Notch-deleted mice (*Rbpj*-deleted) qMuSCs exit the undifferentiated state to directly differentiate and fuse (43), in the D2-depleted model, cells do not exit G_0_ to differentiate, but to transit in a G_Alert_-like state. As such, the D2-depleted phenotype substantially differs from the *Rbpj*-depleted one. In the absence of Notch, a functional D2 and normal TH levels likely allow cell fusion, whereas the absence of D2-TH in the D2-depleted phenotype reduces the Notch signaling and prompts cells to transit in a G_Alert_-like state. We believe the G_Alert_-like state induced by D2 loss represents a specific cell cycle phenotype that is not associated/dependent from the attenuation of Notch signaling. Therefore, the finding that both Notch signaling and G_Alert_-state are regulated by D2-TH does not imply a direct link between the two, but rather suggests that both are part of the pleiotropic effects actioned by TH. However, why do D2-depleted resting MuSCs exit quiescence and remain in a G_Alert_-like state, but without differentiating as observed upon Notch signaling depletion? We believe that the endurance of the G_Alert_-like state in D2-depleted MuSCs may depend on the differentiation defects/delay consequent to insufficient D2-derived TH levels (19). In support of this concept, crucial muscle differentiation transcription factors and differentiation markers, such as all members of the MHC multigene family, respond to TH (46–49).

The enhanced proliferative capacity of D2-depleted cells was confirmed by engraftment experiments, which demonstrated superior engraftment compared with controls. Accordingly, the delayed dystrophic phenotype observed in mdx-D2KO mice is likely attributable to enhanced MuSC expansion, leading to increased myoblast production. The improved functional outcome at the peak of dystrophy is consistent with prior evidence showing that hyperthyroidism exacerbates dystrophic pathology in mdx mice (50), whereas thyroid antagonism delays disease onset and reduces muscle damage in dystrophic animal models (51, 52).

In conclusion, the TH-dependent regulatory mechanism described here provides a strategy to manipulate stem cell behavior using a circulating hormonal signal. While irreversible D2 deletion causes sustained MuSC proliferation with impaired differentiation (19), we show that transient pharmacological D2 inhibition during early regeneration induces MuSC expansion without compromising subsequent differentiation (Figure 7 and Supplemental Figure 6G), resulting in accelerated repair of injured muscle and skin.

Overall, these findings identify TH signaling as a metabolic hub governing stem cell quiescence and suggest that D2 antagonists may represent a therapeutic approach when tissue damage is anticipated, with potential applications in stem cell–based regenerative strategies.

## Methods

Additional details may be found in Supplemental Methods.

### Sex as a biological variable.

Our study examined male and female animals, and similar findings are reported for both sexes. Sex was not considered as a biological variable.

### Animals.

Tg:Pax7^nGFP^, Tg:Pax7^CreERT2^, and R26^mTmG^ mice were provided by Shahragim Tajbakhsh (Institut Pasteur, Université Paris Cité, Paris, France) (43). Dio2^fl/fl^ (53), D2-3xFLAG (30), *mdx* obtained from The Jackson Laboratory (stock 001801) (54), and global D2KO (55) mice were used in this study. Gt (ROSA)26Sor^tm1(Notch1)Dam^/J and C57BL/6 were obtained from The Jackson Laboratory (stock 008159 and 000664, respectively). The Tg:K14-Cre^ERT2^ mouse was previously described (56). Pax7^nGFP^ and Pax7^CreERT2^ were crossed to generate Pax7^nGFP^ Pax7^CreERT2^ mice. Pax7^nGFP^ Pax7^CreERT2^ were further crossed with Dio2^fl/fl^ mice to generate Pax7^nGFP^ Pax7^CreERT2^ Dio2^fl/fl^ mice (referred to as cD2KO). Pax7^CreERT2^ Dio2^fl/fl^ and R26 ^stop-N1ICD-nGFP^ were crossed to generate Pax7^CreERT2^ Dio2^fl/fl^ R26 ^stop-N1ICD-nGFP^ (referred to as cD2KO-N1ICD). Pax7^nGFP^ Pax7^CreERT2^ Dio2^fl/fl^ and R26^mTmG^ were crossed to generate Pax7^nGFP^ Pax7^CreERT2^ Dio2^fl/fl^ R26^mTmG^ mice. K14-Cre^ERT2^ and Dio2^fl/fl^ were crossed to generate K14-Cre^ERT2^ Dio2^fl/fl^ mice (referred to as scD2KO).

All the mice used in this study were males and females between 12 and 16 weeks of age (unless indicated in figures), while the mdx mice used were between 12 and 50 weeks of age, as indicated.

For comparative studies, age and sex were matched in each setting. Animals were genotyped by PCR using tail DNA. Housing conditions were as follows: temperature approximately 72°F (~22°C), 40%–60% humidity, and 14 hour light/10 hour dark daily cycle.

### Cell cultures and reagents.

C2C12 myoblast cells were obtained from ATCC (CRL-1772) and cultured in DMEM (Microgem, AL007-500ML) supplemented with 20% FBS (Microgem, RM10432-500ML), 2 mM glutamine (Gibco, 25030024), 50 IU penicillin, and 50 μg/mL streptomycin (Gibco, 15070063) at 37°C 5% CO_2_. Primary MuSCs (pp6) were isolated from the WT and D2KO mouse model and cultured in DMEM supplemented with 10% FBS, 10% horse serum (HS), 1 ng/mL bFGF (Gibco 13256-029), 5 ng/mL IGF-1, 2 mM glutamine, 50 IU penicillin, and 50 μg/mL streptomycin at 37°C 5% CO_2_. To induce differentiation, cells at 70% confluence were switched from growth medium (20% FBS for C2C12 cells or 10% FBS and 10% HS for pp6 cells) to DMEM supplemented with 2% HS (Gibco, 16050122), 10 μg/mL insulin (Sigma-Aldrich, I2643), and 5 μg/mL transferrin (Sigma-Aldrich, T8158).

### Isolation and culture of single myofibers.

Single myofibers were isolated from the extensor digitorum longus muscle and incubated with 0.1% collagenase type I (Sigma-Aldrich, C9891) in DMEM at 37°C for 60–70 minutes. The muscle was then transferred to a 60 mm Petri dish containing DMEM, and individual myofibers were mechanically dissociated under a dissecting microscope using a heat-polished glass Pasteur pipette treated with HS. Single fibers were transferred to FBS-coated tissue culture dishes in DMEM/F12 (50%) supplemented with 20% FBS and 1% penicillin-streptomycin (Gibco). Fibers were fixed in 4% paraformaldehyde (PFA) (Merck, 1.04005.1000) for 10 minutes immediately or at different time points after plating and stained with specific antibodies. 60–100 myofibers from 4 mice were collected and quantified.

For the experiments with the chemotherapeutic agent AraC (Sigma Aldrich, C1768), myofibers were cultured in medium for 72 hours, incubated with 100 μM AraC for 48 hours, and fixed (day 5). For EdU incorporation assays, myofibers were incubated with 2.5 mg/mL EdU, collected, and fixed with 4% PFA. EdU was detected using the Click-It kit (Invitrogen, C10337) according to the manufacturer’s instructions. EdU incorporation data are expressed as the percentage of EdU^+^ relative to the total number of cells, as measured using DAPI.

### Reserve experiment.

Freshly isolated MuSCs were cultured in growth medium for 3 days and switched to low-serum differentiating medium for another 4 days to induce differentiation as described previously (57). The cells were fixed and stained for Pax7/MyoD (to assess the population of nondifferentiated cells, as reserve cells, Pax7^+^MyoD^–^) and MyHC2 (to assess the induced differentiation); the fusion index (%) was calculated as number of nuclei ≥ 2 in MyHC2/total number of nuclei × 100.

### Western blotting.

MuSCs freshly isolated from cD2KO or control mice were lysed in lysis buffer (50 mM Tris-Cl, pH 8.0, 200 mM NaCl, 50 mM NaF, 1 mM dithiothreitol, 1 mM Na_3_VO_4_, and 0.3% IGEPAL) and protease inhibitor cocktail (Sigma Aldrich, P8340). Cells were boiled for 5 minutes and centrifuged at 845*g* for 10 minutes. Samples were loaded onto 10% SDS-PAGE gels followed by Western blotting. Antibodies used are listed in Supplemental Table 1. Antibody-labeled protein bands were detected using Immobilon Western Chemiluminescent HRP Substrate (Millipore, WBKLS0500). Gel images were analyzed using Image Lab version 5.2.1 software (Bio-Rad).

### RT-qPCR.

Total RNA was extracted from freshly sorted or cultured cells using an RNeasy Micro Kit according to the manufacturer’s instructions (Qiagen, 74004) and then reverse transcribed to cDNA using VILO reverse transcriptase (Invitrogen, 11756050) according to the manufacturer’s instructions. RT-qPCR was performed using an iQ5 Multicolor Real-Time Detector System (Bio-Rad) with SYBR Green Master Mix (Bio-Rad, 1708882). The cyclofilin A gene was used as a housekeeping gene control for ΔCt calculations [ΔCt = (Ct of target gene) – (Ct of housekeeping genes)]. The primer sequences used are listed in Supplemental Table 2. Fold expression values were calculated using the 2^–ΔΔCt^ method, where ΔΔCt = (ΔCt of treatment sample) – (ΔCt of control samples) (with the control value normalized to 1). 3 technical replicates were performed for all qPCR experiments.

### ChIP-qPCR.

C2C12 cells (~2 × 10^6^) or isolated MuSCs (pp6, WT ± rT3 and D2KO) were cross-linked with 1% formaldehyde in culture medium for 10 minutes at room temperature (RT), after which the cells were scraped in RIPA buffer (1× PBS, 1% NP-40, 0.5% sodium deoxycholate, and 0.1% SDS). The cell extract was sonicated, diluted for immunoprecipitation, and incubated with the indicated antibodies or control IgG (rabbit IgG, ab172730) overnight at 4°C. Samples were sequentially washed in low-salt buffer (20 mM Tris-HCl, pH 8.0, 150 mM NaCl, 2 mM EDTA, 0.1% SDS, and 1% Triton X-100), high-salt buffer (20 mM Tris-HCl, pH 8. 0, 500 mM NaCl, 2 mM EDTA, 0.1% SDS, and 1% Triton X-100), LiCl IP wash buffer (100 mM Tris-HCl, pH 7.5, 500 mM LiCl, 1% NP-40, and 1% sodium deoxycholate), and 1× TE (10 mM Tris-HCl, pH 7.5, and 0.1 mM EDTA). All washes were performed at 4°C for 5 minutes. Immunoprecipitates were eluted and de-cross-linked overnight at 65°C. DNA fragments were extracted and RT-qPCR was performed. As a negative control, RT-qPCR was performed using unrelated oligonucleotides, and the presence of equivalent amounts of chromatin in each sample was confirmed by PCR without prior immunoprecipitation (input). The primer sequences are listed in Supplemental Table 2.

### Immunohistochemistry studies.

Dissected muscles were snap-frozen in liquid nitrogen–cooled isopentane, sectioned (7 μm thick), and stained. For IF staining, cells or sections were fixed with 4% PFA, permeabilized with 0.1% Triton X-100, blocked with 0.5% goat serum, and incubated with primary antibody overnight at 4°C. After several washes with PBS, cells were incubated with secondary antibodies for 1 hour at RT. Alexa Fluor 594–, 647–, or 488–conjugated secondary antibodies were used to detect mouse primary antibodies. Images were captured using an Olympus IX51 microscope equipped with Cell*F software or an LSM 980 confocal system equipped with ZEN software (Carl Zeiss). For H&E staining, sections were fixed in 4% PFA for 15 minutes, washed several times, and embedded in hematoxylin (Sigma-Aldrich, GHS116) for 5 minutes and eosin (Sigma-Aldrich, HT110216) for 5 minutes using a standard protocol. For Oil Red O staining (Sigma-Aldrich, O0625), sections were placed in 60% isopropanol followed by incubation in 0.5 g Oil Red O and 1% destrin solution in 98% isopropanol for 75 minutes, and washed in 60% isopropanol and then in water. Sections were counterstained with Mayer’s hematoxylin for 2 minutes. For Sirius Red staining, sections were fixed with 4% PFA for 10 minutes and stained with Sirius Red solution (Abcam, ab150681) for 60–90 minutes at RT, protected from light. After washing in acidified water, sections were fixed in 100% ethanol and dehydrated in 100% xylene. Sections were mounted using EUKITT (Sigma-Aldrich, 03989).

Briefly, TA muscles were dissected and snap-frozen in liquid nitrogen–submerged isopentane. Muscle cross sections were cut at 7 μm and stained with H&E. Muscle sections were imaged at ×20 magnification and quantified using Olympus CellF* imaging software. Each fiber was tracked, and the pixel count was calibrated to obtain the CSA of the muscle.

### Transplantation.

Approximately 1–3 × 10^4^ FACS-isolated MuSCs obtained from 12-week-old TAM-treated Pax7^CreERT2/+^ D2^fl/fl^ R26^mTmG^ or Tg:Pax7^CreERT2/+^ D2^+/+^ R26^mTmG^ mice were resuspended in 20 μL of 1× PBS containing 0.1% BSA. The 8-week-old *mdx* mice were anesthetized by intraperitoneal injection of a ketamine-xylazine cocktail. The donor MuSCs were then transplanted into the TA muscle of the *mdx* mice to assess skeletal muscle regeneration. Transplantation was performed by slowly injecting 10 μL of the donor cell solution into the TA muscle using a 25 μL Hamilton syringe. Each host mouse received transplantation of MuSC cD2KO into the left TA and MuSC control into the right TA. 21 and 40 days after transplantation, frozen TA muscle sections were processed for IF. For transplantation at a longer time point (40 days after xenograft), we treated mice with tacrolimus (an antirejection drug, at 2.5 mg/kg/day) in water starting 11 days after xenotransplantation.

### Hair follicle cycle.

The dorsal back of 3-month-old anesthetized D2KO and control mice was clamped with forceps (58). Mice were harvested at 6 days for anagen, 10 days for catagen, and 60 days for telogen, and dorsal skin was collected for molecular and histologic analysis (59).

### Wound healing.

The dorsal fur of the mice was shaved, and the skin was cleaned with 70% ethanol. The dorsal skin was pulled with forceps, and an 8 mm full-thickness skin wound was created along the midline using a sterile 8 mm circular biopsy punch by pressing through both layers of the skin. Skin wound healing was measured every 2–3 days by anesthetizing the animals and imaging the wounded area. Each wound site was photographed using a Nikon FX-35A camera at the indicated time intervals, and the wound areas were analyzed on the photographs using CellF* software. Changes in wound area are expressed as a percentage of the initial wound area.

### Cell size.

The cell size of MuSCs was evaluated by FACS using forward scatter analysis.

### mtDNA.

DNA was extracted from approximately 50,000 freshly FACS-isolated MuSCs using the QIAamp DNA Micro Kit (Qiagen) according to the manufacturer’s instructions. mtDNA was quantified by qPCR using primers amplifying the cytochrome B region on mtDNA relative to the β-globin region on genomic DNA (Supplemental Table 2).

### RNA-seq.

RNA extraction was performed with the RNeasy Mini Kit (Qiagen). RNA was processed using the NextSeq 500/550 output kit (Illumina) and sequenced using the HiSeq 4000 System (Illumina). FASTQ files containing sequencing reads were aligned to the mouse genome assembly mm10 (NCBI genome assembly GRCh38) using STAR (60) with default options. Genes with a mean of DESeq2-normalized counts (“baseMean”) > 10 were considered to be expressed. Data were analyzed using Rosalind (https://rosalind.onramp.bio/), with a HyperScale architecture developed by OnRampBioInformatics.

### Hanging tests.

To evaluate the muscle functional properties of D2KO-*mdx* compared with *mdx* mice (4–6 weeks of age), we used the 2- and 4-limb hanging tests according to the described protocols (61). For the 2-limb hanging test, the mouse was suspended over a metal wire placed at 37 cm above a cage with soft bedding. After the mouse gripped the wire with its forelimbs, the hanging time was recorded. For the 4-limb hanging test, the grid of a large cage was used, which was located 25 cm above a cage with soft bedding. After the mouse was placed on the grid, it was turned upside down and the hanging time was recorded. The tension (impulse) developed by the animal to maintain itself on the wire or grid against gravity for the longest period of time was analyzed according to: holding impulse (s·g) = body mass (g) × hang time (s). C57BL/6 mice were used as an additional control for comparisons with *mdx* mice.

### rT3 administration in vivo.

rT3 (Sigma-Aldrich, T0281) was administered to mice (C56BL6 mice) via drinking water at a final concentration of 2 μg/mL (or PBS as control) for 10 consecutive days.

### Use of generative artificial intelligence.

ChatGPT (OpenAI) based on GPT-5 was used to edit the Abstract and to prepare the initial version of the graphical abstract (October–November 2025). No artificial intelligence tools were used for data analysis, experimental design, or interpretation of scientific results.

### Statistics.

Significant differences were calculated using ANOVA and 2-tailed Student’s *t* test for independent samples, with *P* < 0.05 considered statistically significant. Statistical tests were performed using GraphPad Prism 7 and are described in more detail in the supplemental materials. In all figures, data are presented as mean ± SEM; significance was determined using a Mann-Whitney test when comparing 2 conditions and 2-way ANOVA when comparing multiple conditions. The sample size for each experiment is given in the corresponding figure legend.

### Study approval.

Mice were housed in a pathogen-free facility at CEINGE Biotecnologie Avanzate Franco Salvatore. Experiments were performed according to the guidelines of the Ministero della Salute and approved by the IACUC (167/2015-PR and 354/2019-PR).

### Data availability.

The RNA-seq raw data generated in this study for WT or cD2KO MuSCs have been deposited in the NCBI GEO under accession code GSE270968. Differentially expressed genes in G_Alert_ state were obtained from the GSE55490 series in GEO. All data values are available in the Supporting Data Values file.

## Author contributions

In vitro and in vivo experiments and figure preparation: MADS, RA, CL, TP, and DDG. Conceptualization: MADS and DS. Formal analysis: MADS, RA, CL, DDG, MD, and C Miro. Investigation: MADS. Observations and scientific interpretation: C Missero. Writing: MADS and DS. Supervision: DS.

## Conflict of interest

The authors have declared that no conflict of interest exists.

## Funding support

- European Research Council under the European Union’s Horizon 2020 Programme—EU FP7 contract THYRAGE (grant 666869).
- PRIN (Projects of Relevant National Interest; project 2022H8LXFR).
- Associazione Italiana per la Ricerca sul Cancro (grant IG 27729 to DS and grant IG 25116 to C Missero).

## Supplementary Material

Supplemental data

Unedited blot and gel images

Supporting data values

## Version 1 03/05/2026

In-Press Preview

## Version 2 05/01/2026

Electronic publication
