# Supplementary material for: Type 2 deiodinase–dependent surge in thyroid hormone controls muscle stem cell quiescence and self-renewal
Source: J Clin Invest. 2026 Mar 5;136(9):e194925. doi: 10.1172/JCI194925 (PMC13132369; doi:10.1172/JCI194925)
Supplement: Supplemental data [file jci-136-194925-s008.pdf]

ARTICLE TITLE:

Type 2 deiodinase–dependent surge in thyroid hormone controls muscle stem cell quiescence and self-renewal.

CONTACT FOR REAGENT AND RESOURCE SHARING

Further information and requests for resources and reagents should be directed to and will be fulfilled by the Lead Contact, Domenico Salvatore (domsalva@unina.it).

DATA AVAILABILITY

The RNA-sequencing raw data generated in this study by wt or cD2KO MuSC have been deposited in the National Center for Biotechnology Information (NCBI) in Gene Expression Omnibus (GEO) under accession code GSE270968. Differentially expressed genes (DEG) in GAlert state were obtained from the GSE55490 series in GEO.

SUPPLEMENTARY FIGURES

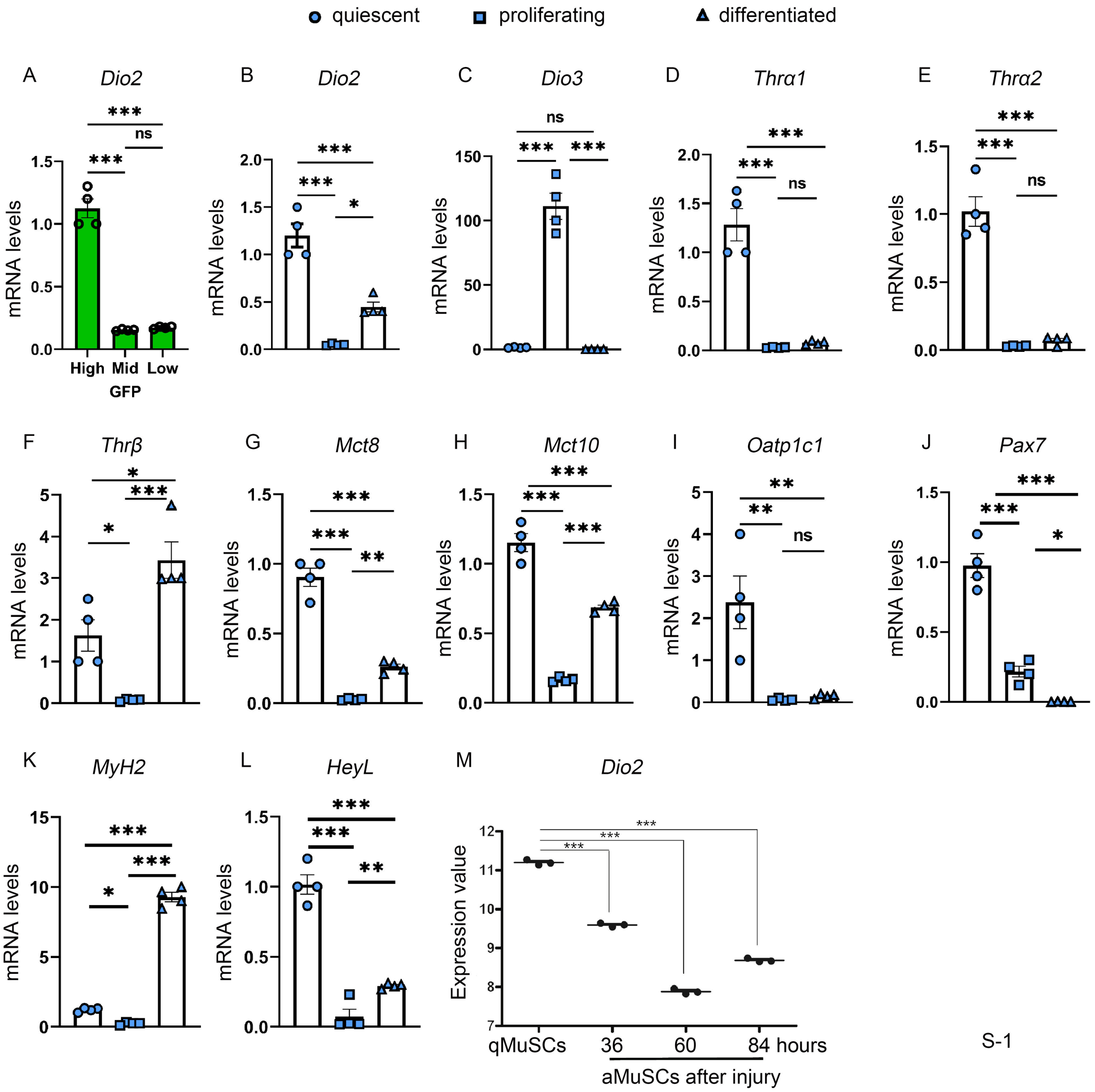

Figure S1. ***Expression of Dio2 and other relevant TH-related genes in MuSCs under different conditions.***

(A) *Dio2* mRNA levels in Pax7-GFP$^{high-mid-low}$ FACS-isolated MuSCs from hindlimb muscles of 3-month-old mice analysed by qRT-PCR (n=4 mice). Expression levels for *Dio2* (B), *Dio3*(C), Thyroid receptors-α1 (*Thrα1*) (D), Thyroid receptors-α2 (*Thrα2*) (E), Thyroid receptors-β (*Thrβ*) (F) Thyroid transporters *Mct8* (G), *Mct10* (H), *Oatp1c1* (I), *Pax7* (J), *MyH2* (K) and *Heyl* (L) genes in quiescent,

proliferating and differentiated conditions were measured by qRT-PCR (n= 4). Data are expressed as mean ± SEM; *p < 0.05, **p < 0.01, ***p < 0.001 by one-way ANOVA. (M) *Dio2* expression score in quiescent and activated MuSCs at different time points after injury within the GSE47177 dataset[52] analyzed with the GEO2R online tool.

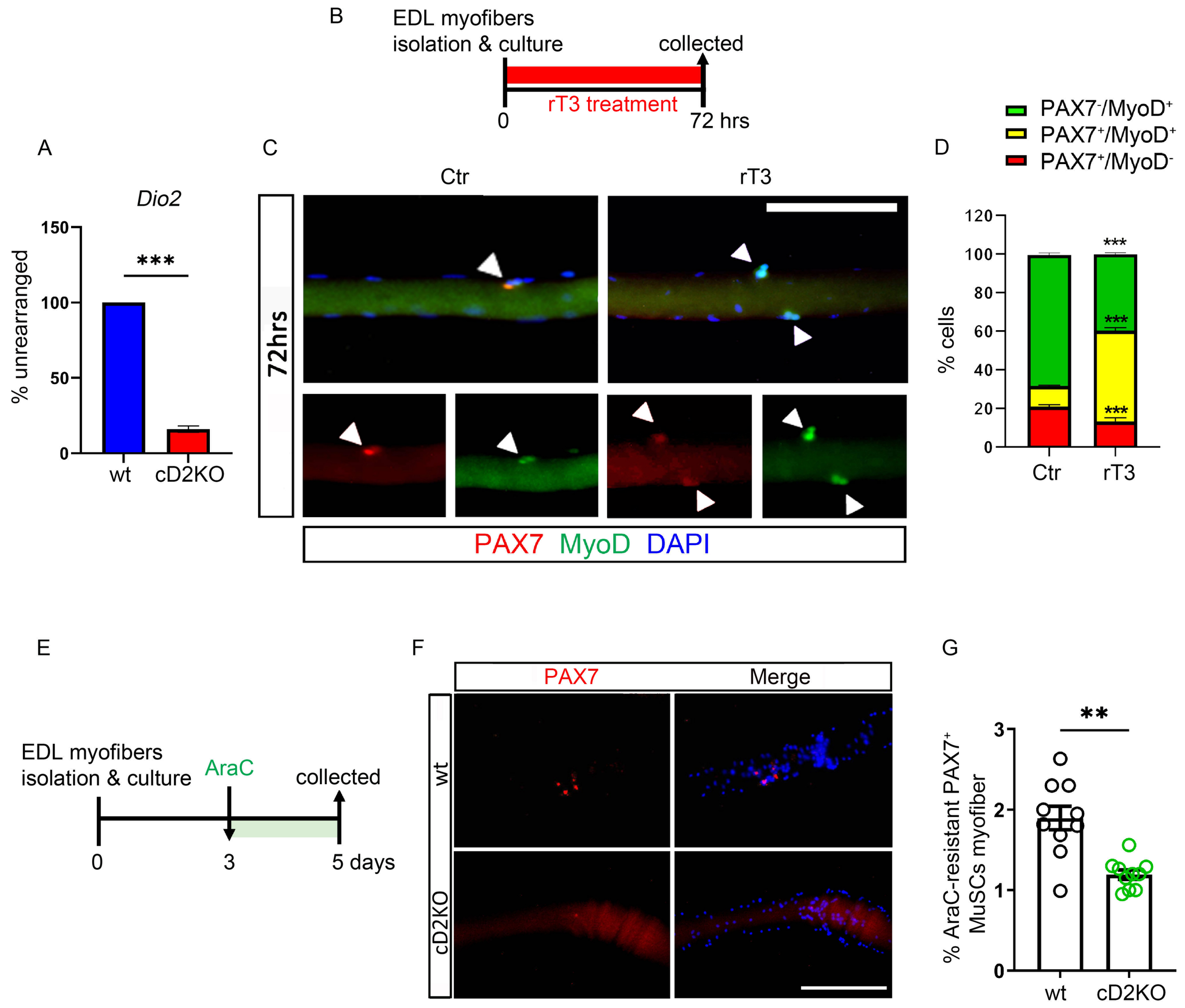

Figure S2. ***D2-depletion sustains MuSCs in the activation phase.***

(A) Percentage of non-rearranged D2 after 5 days of TAM induction. (B) Schematic diagram of the experimental design of C. (C) Representative IF staining of PAX7 (red) and MyoD (green) on isolated myofibers from D2-3xFLAG mice +/- rT3 treatment for 72 hours (Scale bar, 100 µm). (D) Quantification of the percentage of PAX7±/MyoD± cells. n=12 wild type and n=12 rT3-treated mice.

(E) Diagram of the experimental design (F-G). (F) Representative IF staining of PAX7 cells on cultured fibers treated with AraC for 48 hours (Scale bar, 100µm). (G) Percentage of AraC-resistant PAX7+ MuSCs on DAPI. Each point represents the average of at least three technical replicates. n=10 wild type and n=10 cD2KO mice. Data are represented as average ± SEM; *$p < 0.05$, **$p < 0.01$, ***$p < 0.001$ using a Mann-Whitney test when comparing two conditions and ANOVA when comparing multiple conditions.

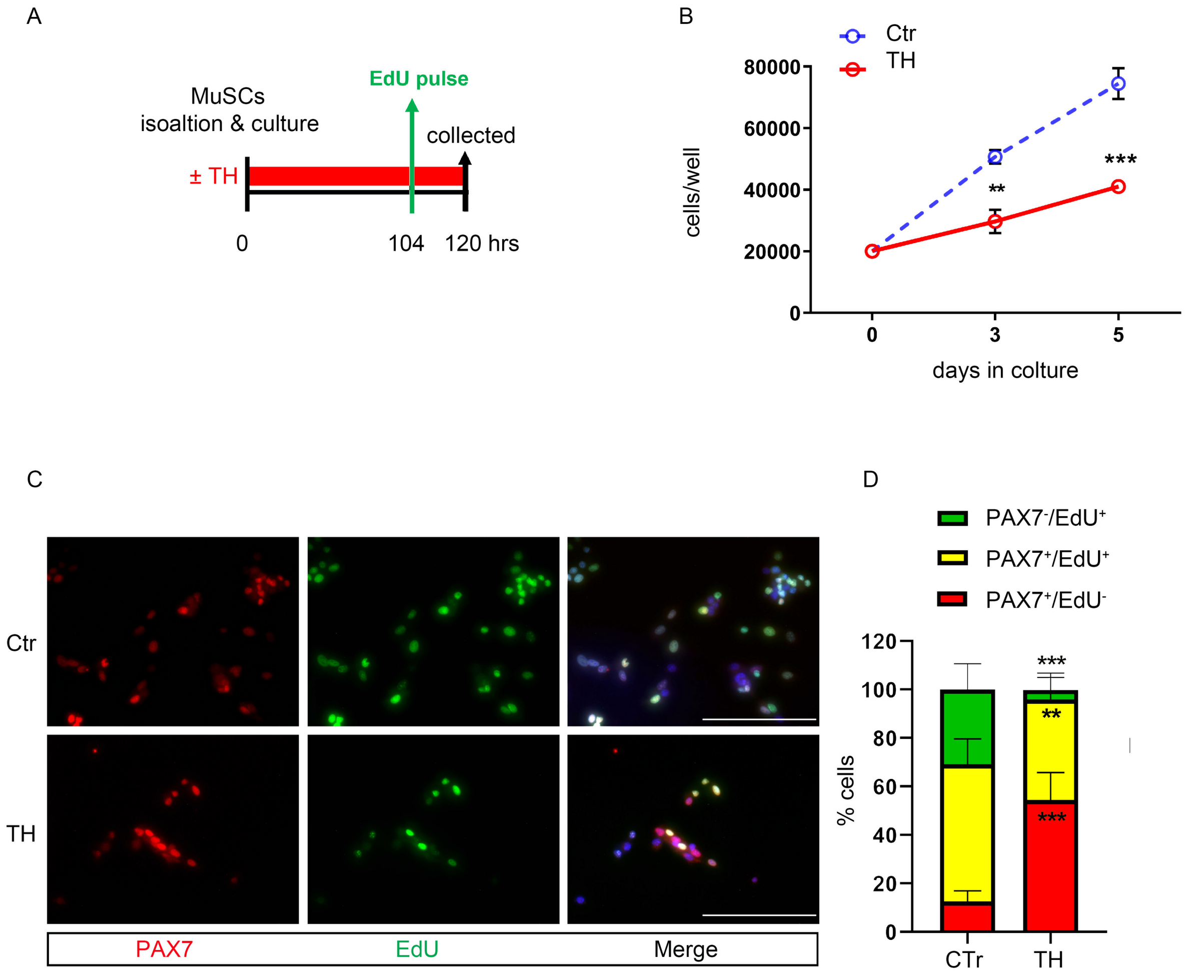

Figure S3. ***TH treatment determines a delay in cell proliferation and activation.***

(A) Schematic of the experiment. FACS-isolated MuSCs were cultured for 5 days under the indicated conditions and then pulsed with EdU for 16 h before harvesting. (B) Growth curve of MuSCs cultured in the absence or presence of exogenous TH (5nM). (C) Representative IF co-staining of PAX7 (red) and EdU (green) (Scale bar, 100μm). (D) Quantification of the percentage of PAX7±/EdU± cells. Results represent the average of 3 independent experiments. Data are presented as mean ± SEM; *p < 0.05, **p < 0.01, ***p < 0.001 by 2-way ANOVA.

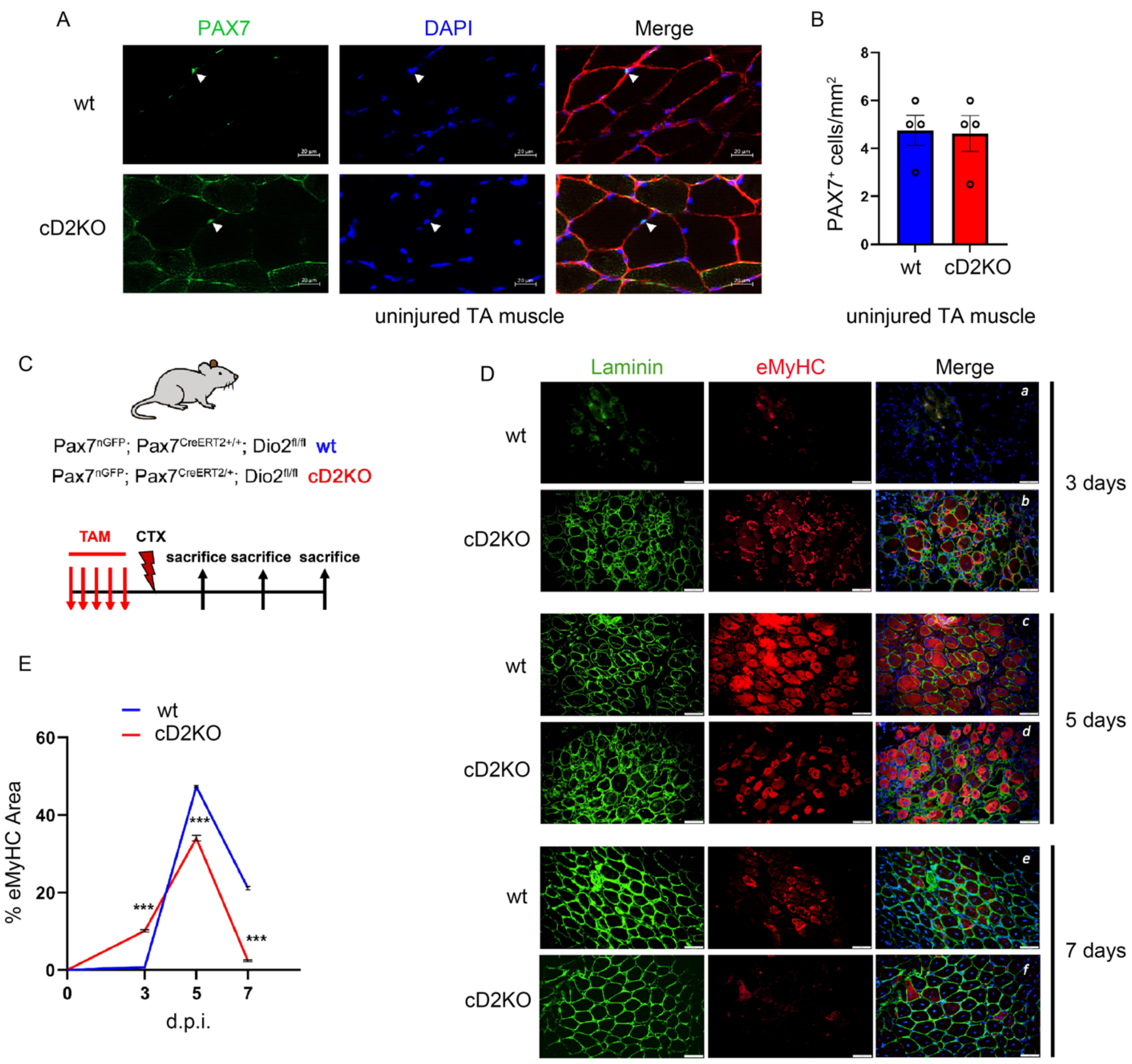

S-4

Figure S4. ***D2 depletion accelerates the early phase of muscle regeneration.***

(A) Representative IF staining of PAX7 (red) and MyoD (green) on uninjured TA muscle (Scale bar, 50 μm).

(B) The number of $PAX7^{+}$ cells per mm^2 in A.

(C) Mouse model used and schematic of the experimental design. cD2KO and wt mice were harvested 3, 5 and 7 days after CTX injection, (n= 4 wt and n= 4 cD2KO for each time point).

(D)Representative immunostaining for Laminin (red) and eMyHC (green) (Scale bars, 50 μm).
(E) Percentage of eMyHC+ area of D. Data are presented as mean ± SEM; *p < 0.05, **p < 0.01, ***p < 0.001 using a Mann-Whitney test when comparing two conditions and 2-way ANOVA when comparing multiple conditions.

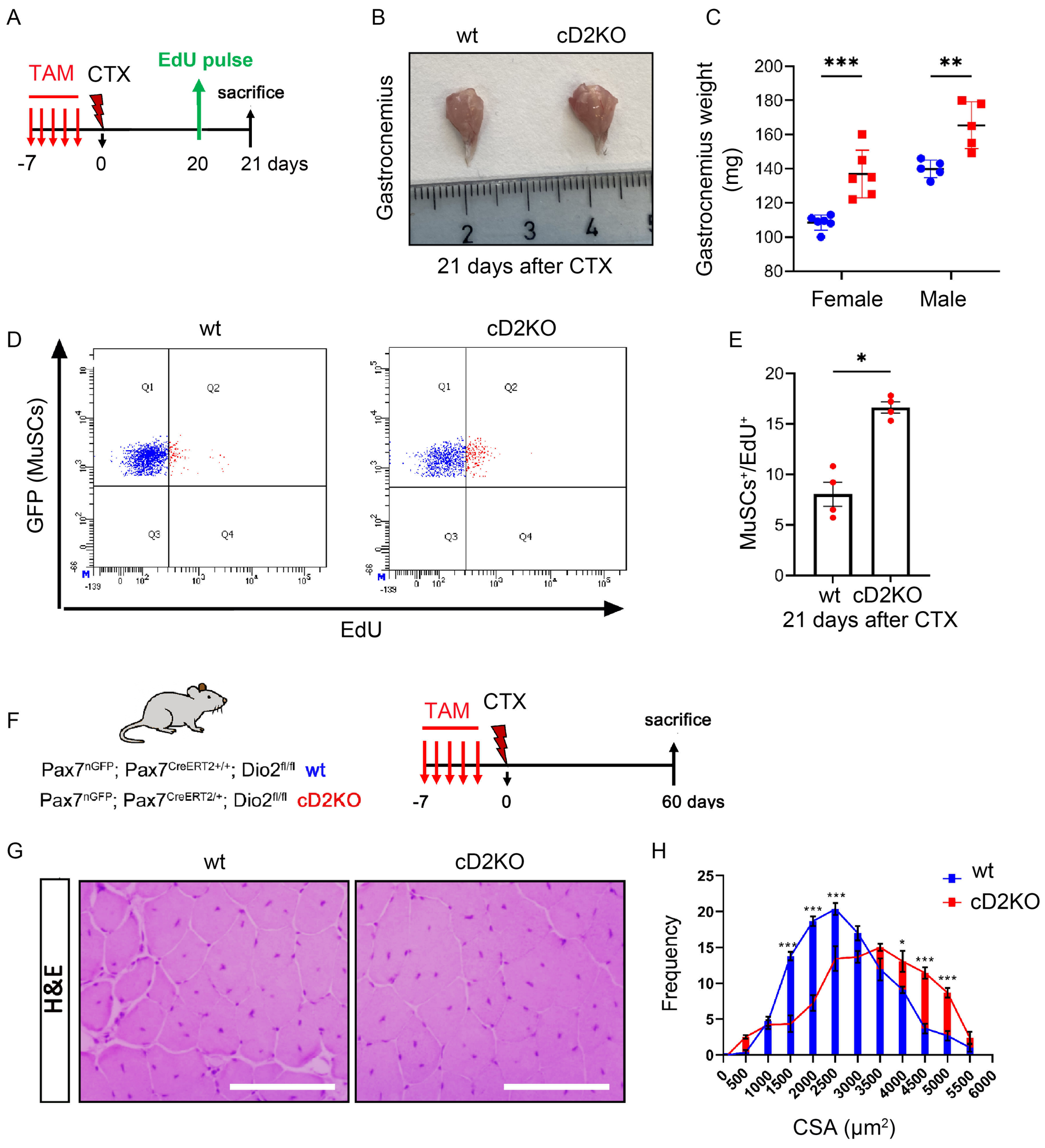

Figure S5. ***Absence of D2 leads, 21 days after injury, to an increased proliferation of MuSCs and muscle hypertrophy.***

(A) Schematic of the experimental design (A-E) in which cD2KO and wt mice were harvested 21days after CTX injection and 1 days after EdU. (B) Image of gastrocnemius muscle from wt and cD2KO of A. (C) Muscle gastrocnemius weight of A. (D) Representative FACS plots for MuScs EdU of wild type and cD2KO mice of A.

(E) Percentage of MuSCs EdU+ in D. Bars are presented as mean values ± SEM (n=4 independent experiments with n=4 wild type and n=4 cD2KO mice each).

(F) Mouse model used and schematic of the experimental design F-H in which cD2KO and wt mice were harvested 60 days after CTX injection. (G) H&E staining of the TA sections (Scale bar, 100 μm). (H) Quantification of the cross-sectional area of TA sections. Data are presented as mean ± SEM; $*p < 0.05$, $**p < 0.01$, $***p < 0.001$ using a Mann-Whitney test when comparing two conditions and 2-way ANOVA when comparing multiple conditions.

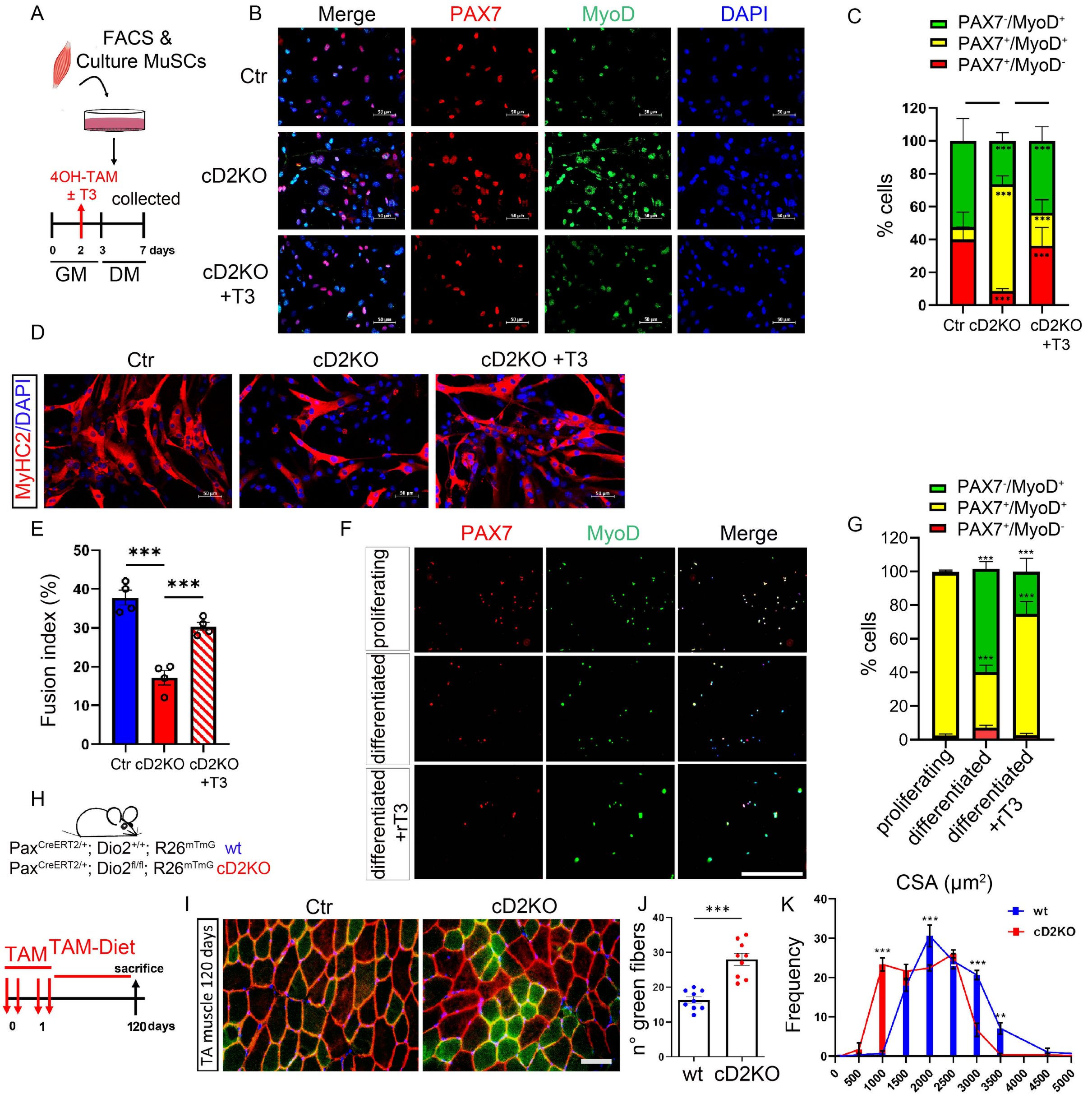

S-6

Figure S6. ***D2-depletion results in an increased number of activated MuSCs and a progressive reduction of the stem cell pool.***

(A) Schematic design of the "reserve experiment" (A-E). MuSCs isolated from cD2KO and wild type mice were plated and cultured in growth medium for 3 days, treated with 1 μM 4OH-TAM and/or 5

nM T3 as indicated, and harvested 4 days later. Two separate experiments were conducted with n=5 wild type and n=5 cD2KO mice each.

(B) Representative immunostaining of PAX7 (red) and MyoD (green) (Scale bar, 50 µm).

(C) Percentage of PAX7±/MyoD± cells.

(D) Representative immunostaining for MyHC2 (red) and DAPI (blue) (Scale bar, 50 µm).

(E) Fusion Index was calculated by dividing the number of nuclei in $MyHC^{+}$ myotubes with ≥2 nuclei by the total number of nuclei analysed (n >10).

(F) Representative IF co-staining of PAX7 (red) and MyoD (green) in proliferating, differentiated and rT3-treated differentiated MuSCs (scale bar, 200 µm). (G) Quantification of the percentage of PAX7±/MyoD± cells in each group in F. Bars represent the average of at least three technical replicates. n=9 mice per group each.

(H) Mouse model used and diagram of the experimental design in I-K, (n=9 wild type and n=9 cD2KO mice). (I) Representative green epifluorescence on TA muscle sections following 120 days after TAM-induction (Scale bar, 200 µm). (J) Percentage of green fibers in I. (K) Quantification of green fiber cross-sectional area.

Data are presented as mean ± SEM; *p < 0.05, **p < 0.01, ***p < 0.001 using a Mann-Whitney test when comparing two conditions, and 2-way ANOVA when comparing plus conditions.

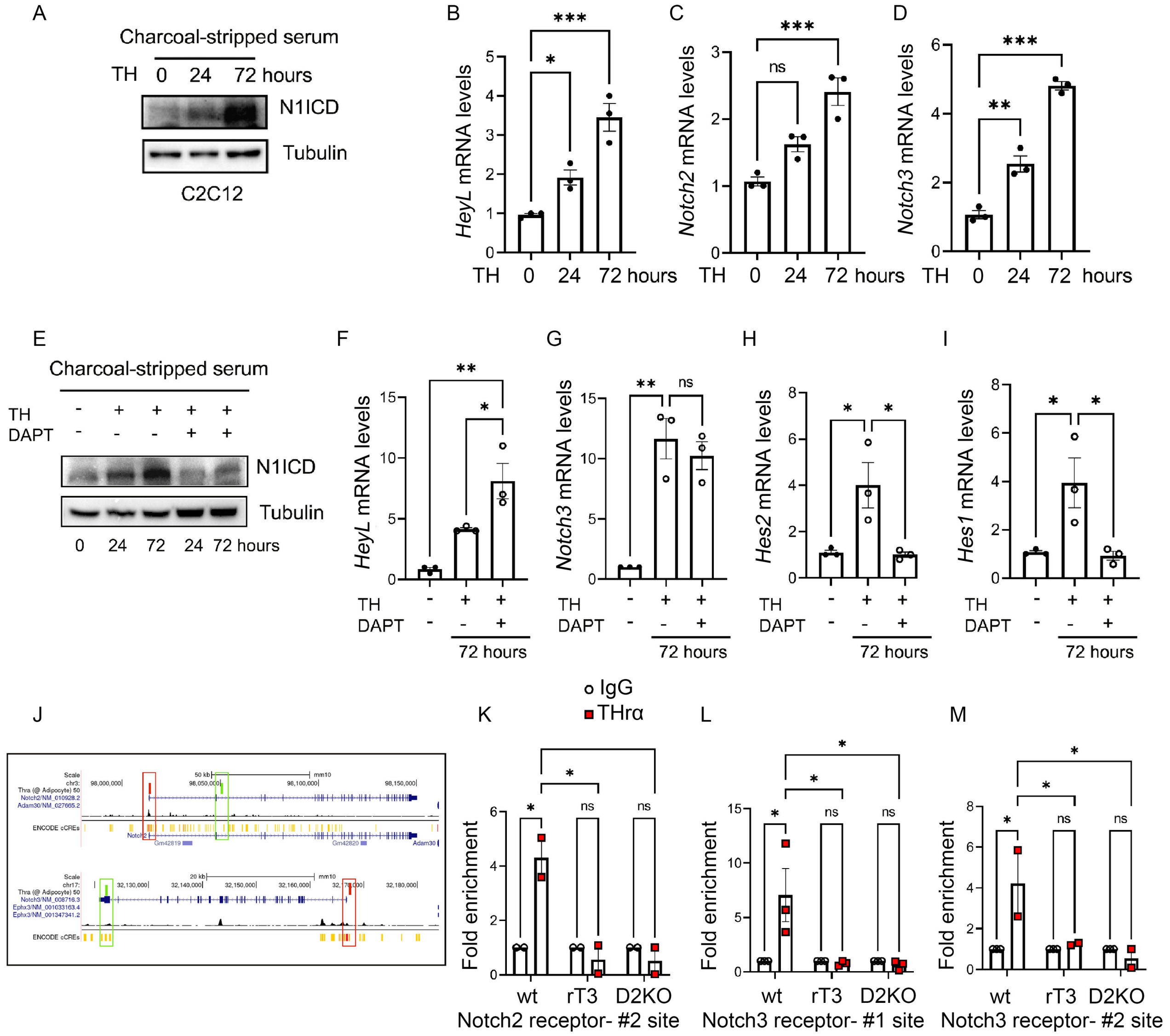

Figure S7. Thyroid hormone regulates relevant components of the canonical Notch pathway by direct and undirect mechanisms.

(A) Protein levels of N1ICD measured by Western blot from C2C12 cells treated with TH for 24 and 72 hours. Tubulin served as a loading control.

(B-D) mRNA levels of *HeyL*, *Nocth2* and *Notch3* receptors in C2C12 cells treated with TH for 24 and 72 hours as indicated. Gene expression was determined by RT-qPCR. Data are presented as mean values ± SEM (*n* = 3 biologically independent samples).

(E) Protein levels of N1ICD measured by Western blot from C2C12 cells treated with TH and/or DAPT for 48 and 72 hours. Tubulin served as a loading control.

(F-G) mRNA levels of *HeyL*, *Notch3, HES2 and HES1* in C2C12 cells treated with TH and/or DAPT for 24 and 72 hours as indicated. Gene expression was determined by qRT-PCR. Data are presented as mean values ± SEM (*n* = 3 biologically independent samples).

(J) Genomic localization of candidate THRα binding sites at the Notch2 and Notch3 loci. Genome browser views of the mouse Notch2 (top, chr3) and Notch3 (bottom, chr17) genomic regions are shown. Gene annotations are indicated in blue and ENCODE candidate cis-regulatory elements (cCREs) are displayed. For each locus, two putative THRα binding regions were selected for analysis and are highlighted by colored boxes. The red box (site #1) corresponds to a promoter-proximal region located upstream of the transcription start site. The green box (site #2) marks a distal regulatory region overlapping a THRα ChIP-seq peak previously identified in brown adipocytes (black track; GEO accession GSM6051365).

(K-M) ChIP-qPCR using THR-alpha and isotype IgG control antibodies on proximal enhancer and promoter regions of Notch2 in pp6 cells. Data are normalized to input chromatin (n=3 biologically independent samples). Data are presented as mean ± SEM; *$p < 0.05$, **$p < 0.01$, ***$p < 0.001$ using a Mann-Whitney test when comparing two conditions, and 2-way ANOVA when comparing plus conditions.

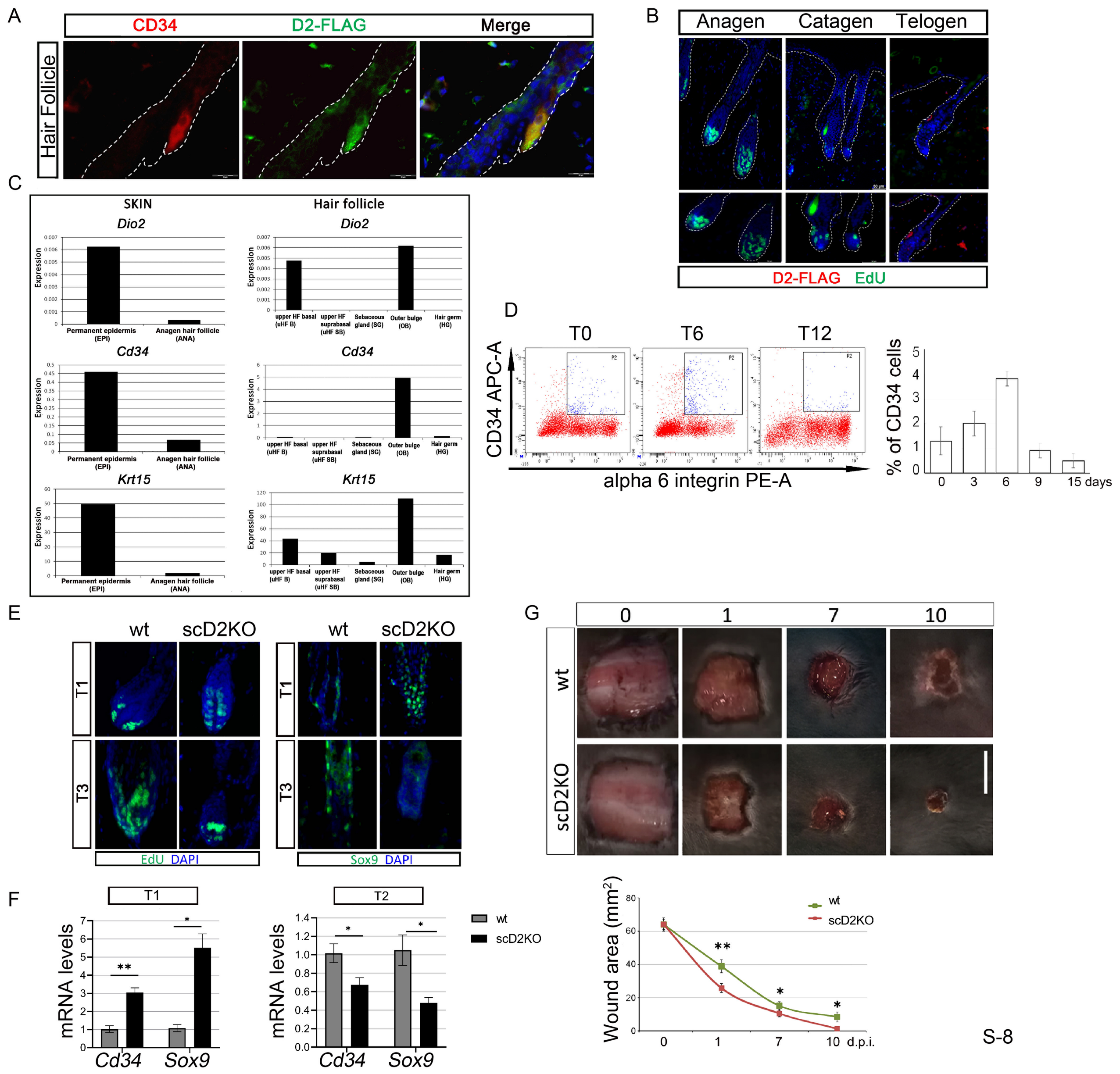

Figure S8. ***Analysis and functional characterization of D2 expression in skin.***

(A) Representative IF co-staining of CD34 (red) and D2-FLAG (green) in the hair follicle (Scale bar, 40 μm). (B) Representative IF of EdU- and D2- positive (green) cells during the hair follicle cycle (scale bar 50 μm (top), 20 μm (bottom)). (C) In silico data analysis of D2 expression from the publicly available dataset (http://kasperlab.org/mouseskin) in mouse skin and hair follicle cycle from single cell RNA sequencing of mouse epidermis. (D**)** Percentage of α6-integrin^{+}/CD34^{+} cells were evaluated by FACS analysis from the epidermis of scD2KO at 0 (T0), 6 (T6) and 12 days (T12) after TAM-induced D2 depletion (right). (E) EdU/DAPI and Sox9/DAPI IF co-staining in the hair follicle after one (T1) and three (T3) consecutive shavings in scD2KO and Ctr mice. (F) CD34 and Sox9 mRNA

levels were measured by qRT-PCR in the hair follicle after one (T1) and three (T3) consecutive shaves in scD2KO and Ctr mice. (G) Wound healing experiment in scD2KO and Ctr mice after 7 days of TAM treatment. Representative images of the area at 0, 1, 7 and 10 days after wounding and its quantification (bottom). Wound area was calculated using Cell*F Olympus Imaging Software (Scale bar, 1 cm). Data are presented as mean ± SEM; *$p < 0.05$, **$p < 0.01$, ***$p < 0.001$ using t-student test when comparing two conditions and ANOVA when comparing plus conditions.

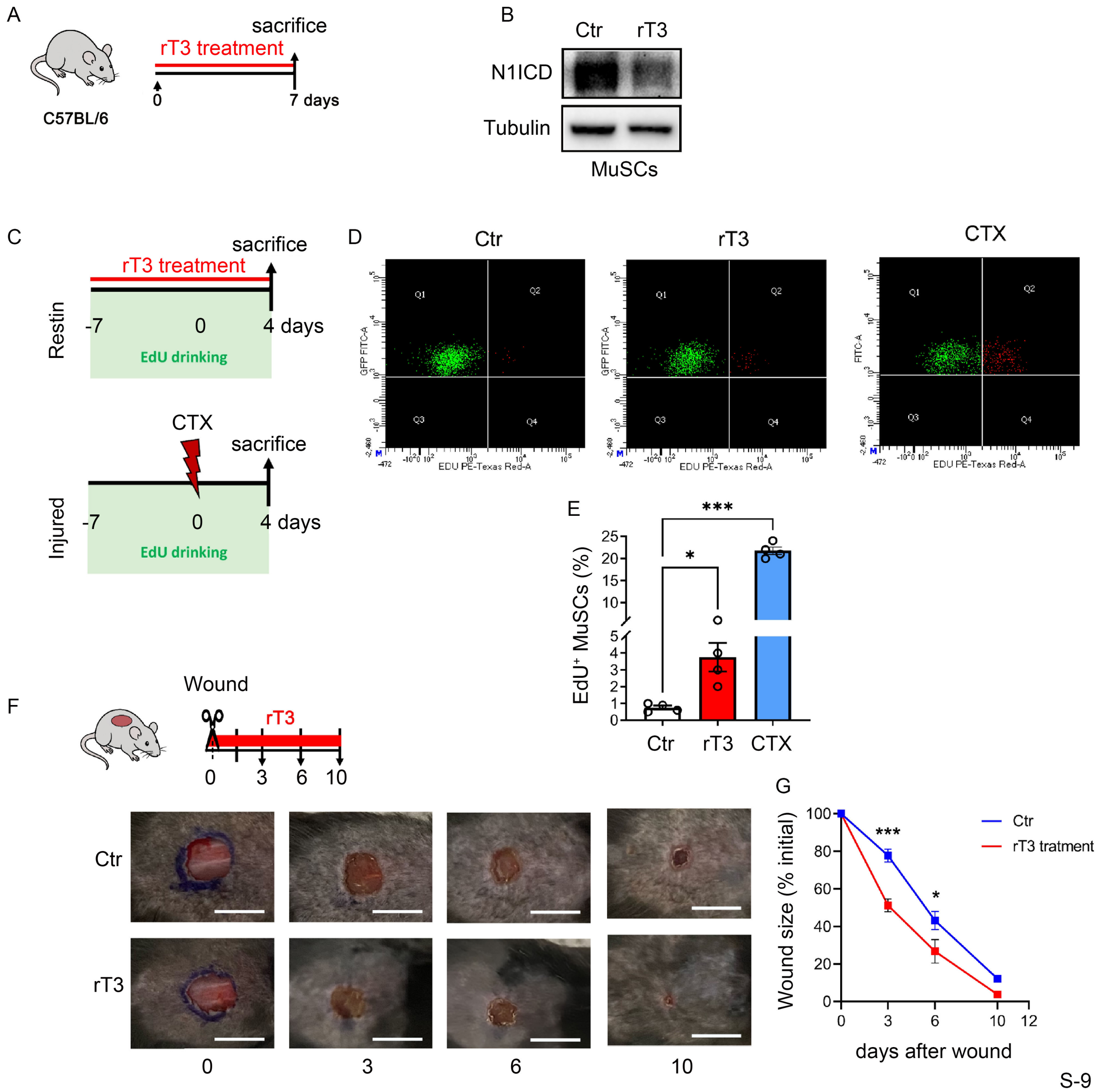

Figure S9. Effects of rT3 treatment in mice and its similarity with genetic D2 depletion.

(A) Schematic diagram illustrating the experimental design in rT3 treated C57BL/6 mouse.

(B) Protein levels of N1ICD measured by Western blot from FACS-isolated MuSCs from resting Ctr and rT3 treated mice. Tubulin served as a loading control.

(C) Schematic representation of the in vivo EdU incorporation experiment in rT3 treated mouse.

(D) Representative FACS plots for EdU/Pax7-GFP of MuSCs isolated from resting tibialis anterior muscle of rT3and Ctr mice (upper panels) and injured muscles of Ctr mice (lower panels).

(E) Percentage of EdU+/MuSCs in D.

(F) Schematic representation and representative images of the wound healing experiment at wound creation (0) and after 3, 6, and 10 days.

(G) Quantification of wound closure as expressed in the mean percentage decrease compared to the initial wound size (scale bar, 1 cm). Bars are presented as mean values ± SEM (n=4 mouse per group). Data are presented as mean ± SEM; *$p < 0.05$, **$p < 0.01$, ***$p < 0.001$ using ANOVA.

SUPPLEMENTAL METHODS

Tamoxifen treatment of mice and *in vivo* EdU incorporation assay

Tamoxifen (TAM) (Sigma Aldrich, T5648) was dissolved in corn oil (Sigma Aldrich, C8267)/10% ethanol (Carlo Erba, #4146052) at a concentration 10 mg/mL and stored at 4°C. For inducible Cre^{ERT2} experiments, mice were injected intraperitoneally (IP) with 2 mg TAM (~ 200 µL, 80 mg/mL) for five consecutive days. For *in vitro* experiments, 4-hydroxy-N-desmethyltamoxifen (4OH-TAM, active metabolite) was used at a final concentration of 1µM. For the *in vivo* EdU incorporation assay, EdU (Sigma Aldrich, #900584) was prepared at 20 mg/mL in saline solution and 6 mg EdU (~ 300 µL) was injected IP into mice along with TAM induction. EdU was detected using the Click-iT EdU Assay (Invitrogen, C10337) according to the manufacturer's instructions.

Fluorescence Activated Cell Sorting

MuSCs were isolated from Tg:Pax7-nGFP mice by fluorescence-activated cell sorting (FACS) as previously described [36]. Briefly, limb muscles from adult mice (3-4 months old) were dissected and minced in ice-cold DMEM. Samples were then digested with a mixture of 0.1% collagenase A (Sigma Aldrich, C9891), 0.2% Dispase II (Roche, #04942078001) and 2.5mM $CaCl_2$ for 30 minutes at 37°C in a shaking water bath. After digestion, DMEM was added to the muscle suspension and filtered, through a 70-µm cell strainer (Corning, #431751), and then centrifuged at 50g for 10 minutes at 4°C to remove large tissue fragments. The supernatant was collected and washed twice by centrifugation at 600 g for 15 minutes at 4°C. Before FACS, the final pellet was resuspended in cold DMEM and 1% penicillin-streptomycin supplemented with 2% FBS, and the cell suspension was filtered through a 30-µm strainer (BD Bioscience, #340598). FACS was performed using FACS Aria IIIu (Becton Dickinson) by gating for GFP fluorescence. MuSCs were cultured in 1:1 DMEM:MCDB (Gibco, 10372019) containing 20% FBS, 1% penicillin-streptomycin 2% ultroser™G (PALL-life sciences,

#15950-017). Cells were plated on Matrigel (BD Biosciences; catalog #354234) or harvested in lysis buffer for RNA or protein extraction.

For cell-sorted epithelial skin, dorsal skin was removed from mice and treated with trypsin 0.25% (Sigma Aldrich, T4799) overnight after removal of adipose tissue. The epidermal layer was separated from the dermis, minced, and incubated with trypsin 0.25% for 7 minutes at 37°C. After digestion, FBS was added to inactivate the trypsin. Cells were filtered through a 70-µm cell strainer. Cell sorting was performed using APC-anti-mouse CD34 (Biolegend, #119310) and PE-rat anti-human integrin alpha-6 (BD Pharmingen, #555736) (Table S1), after incubation for 1 hour on ice. Fluorescence-activated cell sorting analysis was performed using FACS Aria IIIu (Becton Dickinson).

Declaration of Artificial Intelligence and Large Language Models statement.

ChatGPT (OpenAI) was used only to assist with English editing and to prepare the initial version of Graphical abstract. No AI tools were used for data analysis, experimental design, or interpretation of scientific results.

Table S1

Antibodies					
**Name**	**Host**	**Source**	**Cat. number**	**Dilution**	**Application**
ANTI-FLAG M2	Mouse	Sigma-Aldrich	F3165	1:1000	IF, WB
(APC)-anti-mouse CD34	Mouse	Biolegend	119310	1:500	IF; FACS
Alexa Fluor 488 goat anti-mouse IgG	Goat	ThermoFisher Scientific	A-11001	1:500	IF
Alexa Fluor 594 donkey anti-mouse IgG	Donkey	ThermoFisher Scientific	A-21203	1:500	IF
Alexa Fluor 594 donkey anti-rabbit IgG	Donkey	ThermoFisher Scientific	A-21207	1:500	IF
AMPK-alpha (23A3)	Rabbit	Cell Signaling	#2603	1:500	WB
Anti-Thyroid Hormone Receptor antibody [C3]	Mouse	Abcam	ab2743	1:1000	ChIP
Cleaved Notch1 (Val1744) (D3B8)	Rabbit	Cell Signaling	# 4147	1:500	WB
D3 (718)	Rabbit	Home made[66]		1:500	IF
GFP (B-2)	Rabbit	Santa Cruz Biotechnology	sc-9996	1:500	IF
MyoD	Rabbit	Santa Cruz Biotechnology	sc-304	1:500	IF
MyoD	Rabbit	Cell Signaling	#13812	1:200	IF
p44/42 MAPK (Erk1/2)	Rabbit	Cell Signaling	#4695	1:500	WB
Pax7 (PAX7)	Mouse	Developmental Studies Hybridoma Bank		1:20	IF
PE-rat anti human alpha-integrin	Rat	BD Pharmingen	555736	1:500	FACS
Phospho AMPK-alpha (T172) (40H9)	Rabbit	Cell Signaling	#2535	1:500	WB
Phospho mTor (Ser2448)	Rabbit	Elabscience	E-AB-20929	1:600	IF
Phospho S6K1 (T3089)	Rabbit	Abcam	2571	1:500	WB
Phospho-S235/6 S6 ribosomal protein	Rabbit	Cell Signaling	#2211	1:500	WB
α Tubulin	Mouse	Santa Cruz Biotechnology	sc-8035	1:1000	WB

Anti-Laminin-2	Rat	Sigma-Aldrich	L0663	1:500	IF
Anti-Laminin	Rabbit	Sigma-Aldrich	L9393	1:500	IF
Cleaved Notch1 (D1E11)	Rabbit	Cell Signaling	# 3608	1:1000	WB

Table S2

Oligonucleotides used for real-time PCR		
**Gene**	**Forward primer (5' → 3')**	**Reverse primer (5' → 3')**
Cyclophilin A (CypA)	CGCCACTGTCGCTTTTCG	AACTTTGTCTGCAAACAGCTC
Dio2	CTTCCTCCTAGATGCCTACAAAC	GGCATAATTGTTACCTGATTCAGG
Dio3	CCGCTCTCTGCTGCTTCAC	CGGATGCACAAGAAATCTAAAAGC
Mct10	CCATCGTGAGTGTCTTCACGGACAT	GGAGCAGGATTGTGAAGACGCTGC
Mct8	TTCGGCTGGATAGTGGTGTTTGCA	ATCATGCCCATAGCGAGGGCTC
Oatp1c1	GGCTCATCAACATTCCTGCAGTGG	CAGCAAGACAAGCCGACACATAGG
Thra1	ACCACCGCAAACACAACATT	CATTCCGAGAAGCTGCTGTC
Thra2	CCTGGATGACACGGAAGTGGCT	GGGCACTCGACTTTCATGTG
Thrb	CACAGGGTACCACTATCGCTGC	CAGCACCAAGTCTGTTGCCATGC
Cd34	CTCTGCCTGATGAGTCTGCT	TGGTGTGGTCTTACTGCTGT
Sox9	AGGGCTACGACTGGACGCTGGTG	TGTAATCGGGGTGGTCTTTCTTGTGCT
Myod1	GACCTGCGCTTTTTTGAGGACC	CAGGCCCACAGCAAGCAGCGAC
Myog	TTGCTCAGCTCCCTCAACCAGGA	TGCAGATTGTGGGCGTCTGTAGG
Ck (Ckm)	GACCTCAGCAAGCACAACAA	CCTGGATGATGGGATCAAAC
HeyL	TCTTGCAGATGACCGTGGAT	GCATAGCTCTTGAGGTGGGA
Hes2	CTGCCCAGAAGAGTTGAGGA	AGCTATTCAAAGGCCCAGGT
Rbpj	ACAGACAAGGCCGAGTACAC	ACATCCCCAAACCACACTCT
Notch1	GGTCGCAACTGTGAGAGTGA	TTGCTGGCACATTCATTGAT
Notch2	GCAGGAGCAGGAGGTGATAG	GCGTTTCTTGGACTCTCCAG
Notch3	CTCATCAGGAACCGCTCCAC	TCCCGAGCGGCCAAGAACAG
Notch4	ATCCCTCTAACCTGCATCCC	GACCCTCAGAGTCAGGGACA
Pax7	ACCCTGATGCATGGTTGATGG	GTCTGGTTCAGTAACCGGCGTG
Desmin (Des)	TACACCTGCGAGATTGATGC	ACATCCAAGGCCATCTTCAC
MyHC2	CCATTCAGAGCAAAGATGCAGG	GCATAACGCTCTTTGAGGTTG
Hes1	TTCTTAAGTGCATCCAAAATCA	GCCTCTGAGCACAGAAAGTCA
**Oligonucleotides for mitochondrial DNA**		
Cytochrome B	CATTTATTATCGCGGCCCTA CATTTATTATCGCGGCCCTA	TGTTGGGTTGTTTGATCCTG
b-Globin	GAAGCGATTCTAGGGAGCAG	GGAGCAGCGATTCTGAGTAGA

Oligonucleotides used for ChIP analysis		
**Gene**	**Forward primer (5' → 3')**	**Reverse primer (5' → 3')**
5' Notch2	CTCCCCATGCGGATCTGCTC	GACGAGGCAGTTTGGAAGAC
5' Notch3	GGTTTGGAGTCAGGGGTGTT	CCTCGGTACACTCAGGTCTG
Notch2	GTCTCTCCAGCCCAGAAGTT	AGGCCTCCTTTTCCAAACCT
Notch3	AGCATACTTCAGAGGCCAGG	CAGTCTTCTCAGCCCTGGAA
