# Supplementary figures and images for: Type 2 deiodinase–dependent surge in thyroid hormone controls muscle stem cell quiescence and self-renewal
Source: J Clin Invest. 2026 Mar 5;136(9):e194925. doi: 10.1172/JCI194925 (PMC13132369; doi:10.1172/JCI194925)

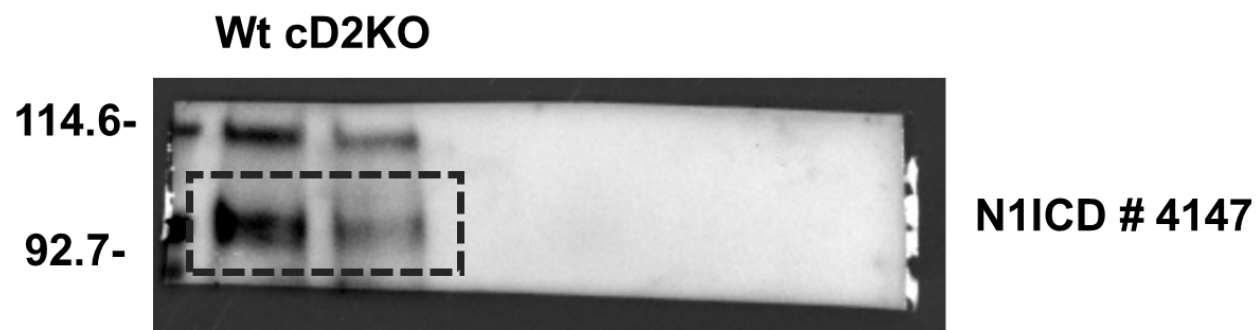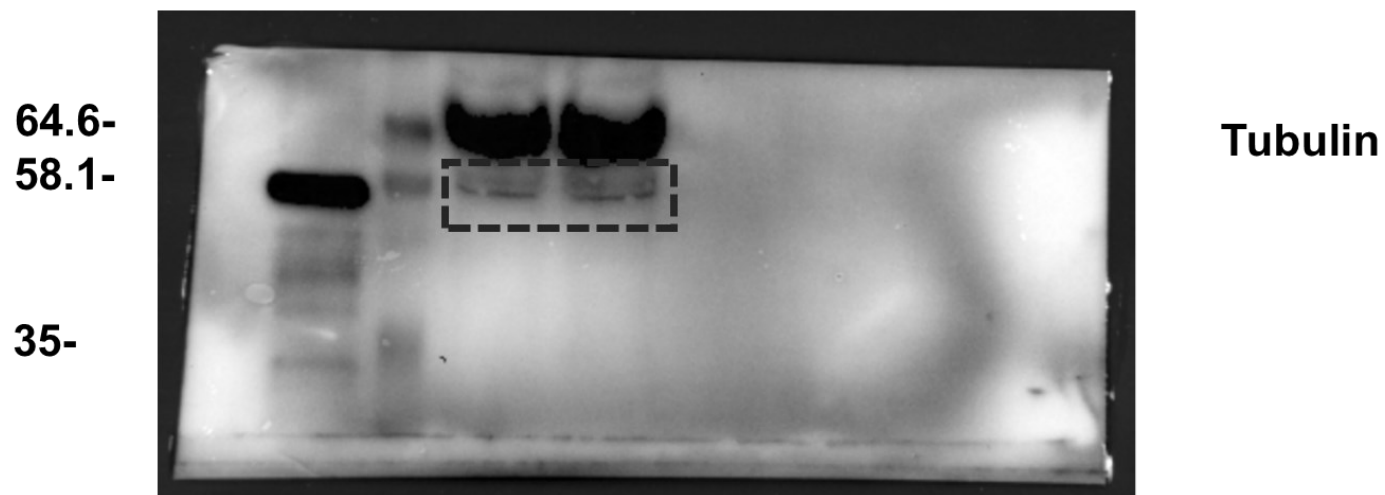

**Figure 4E**

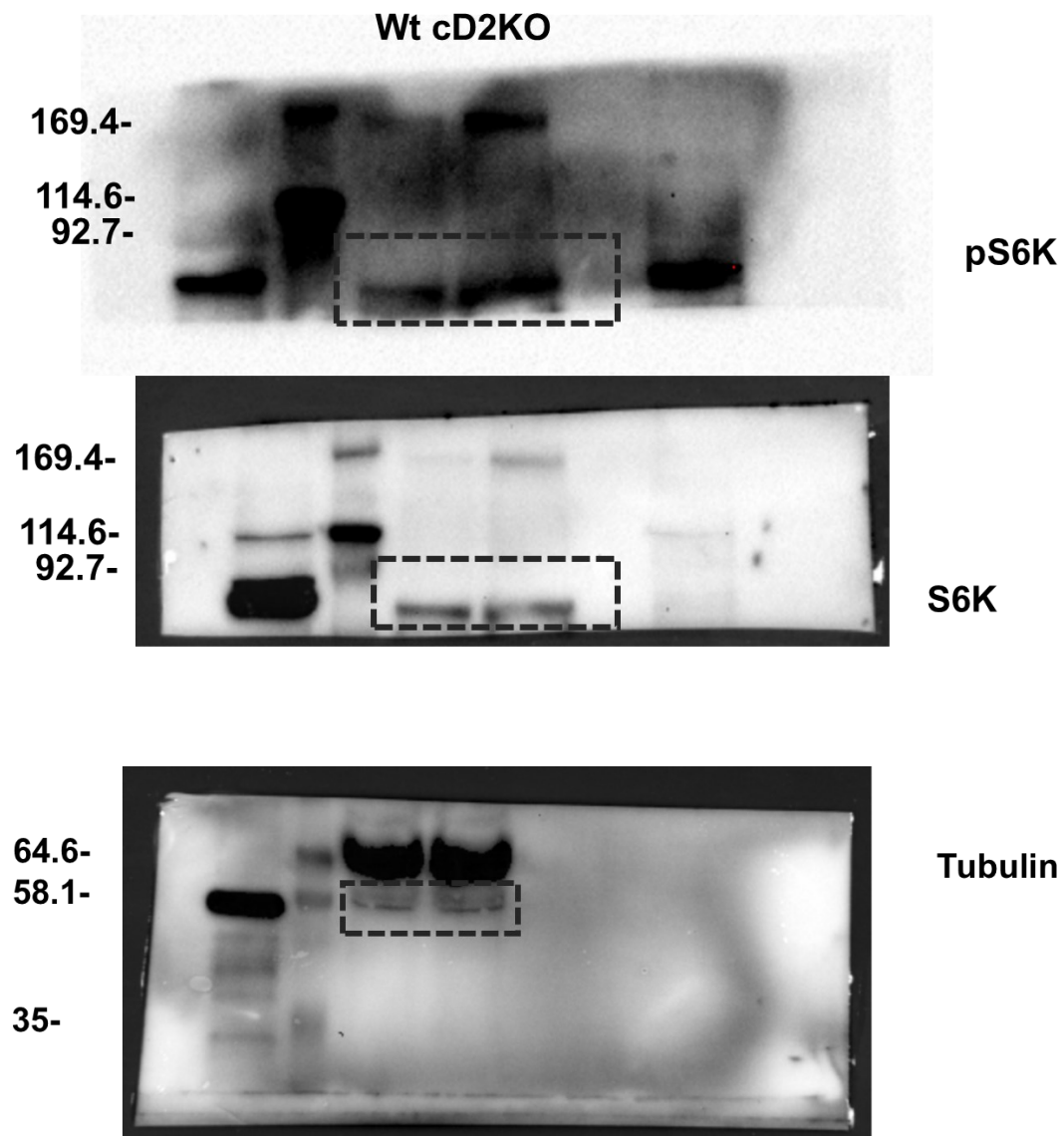

**Figure 5F**

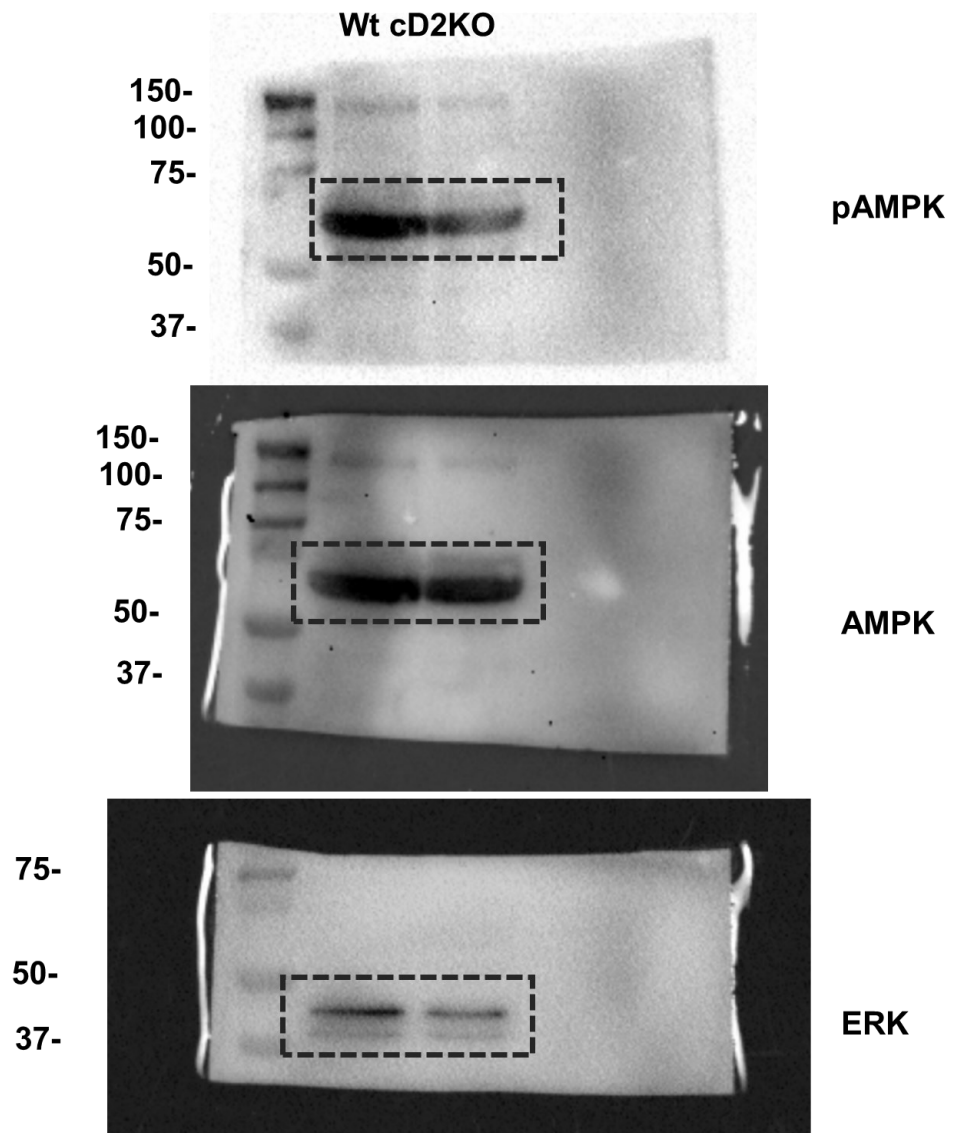

Figure 5G

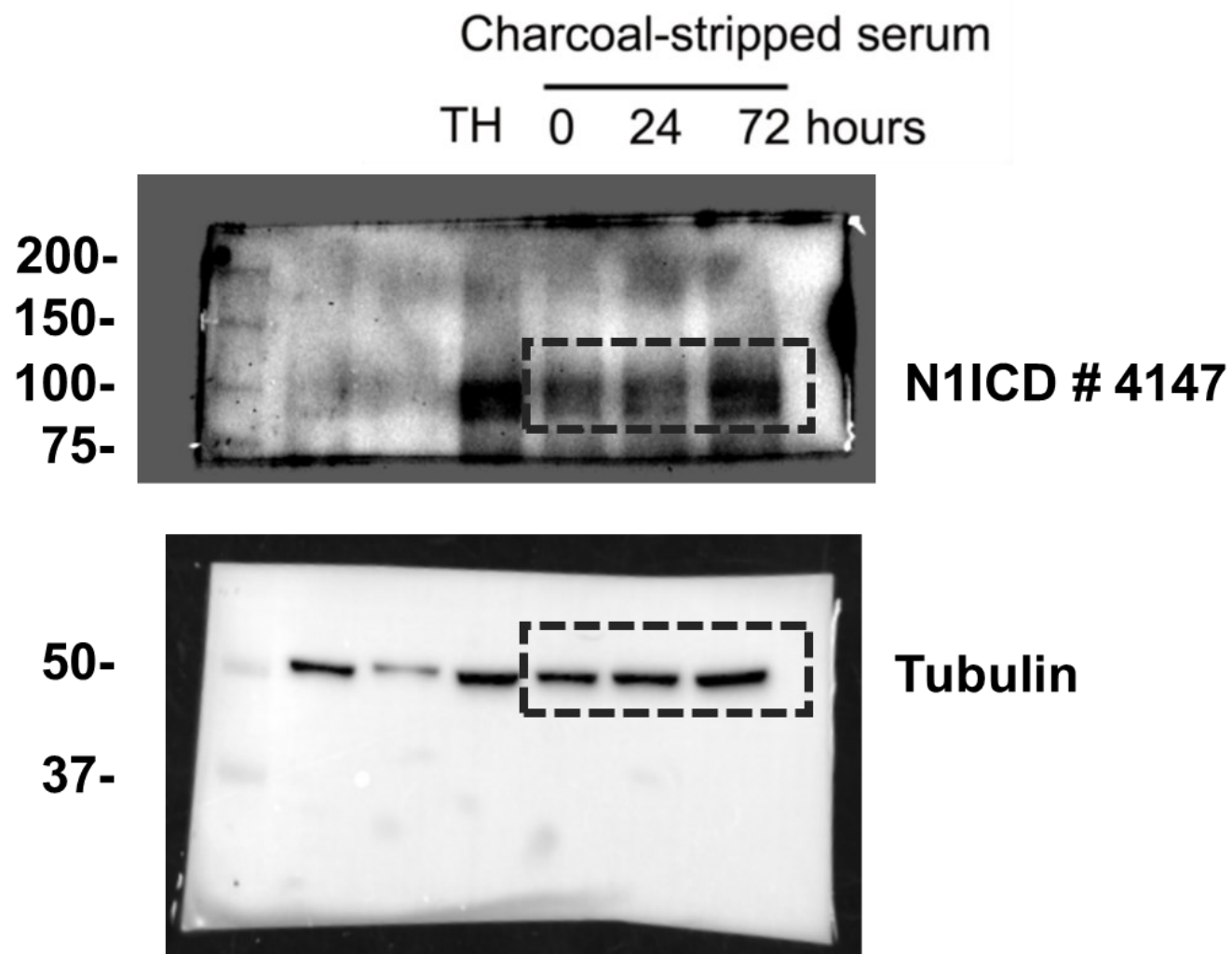

**Figure S7A**

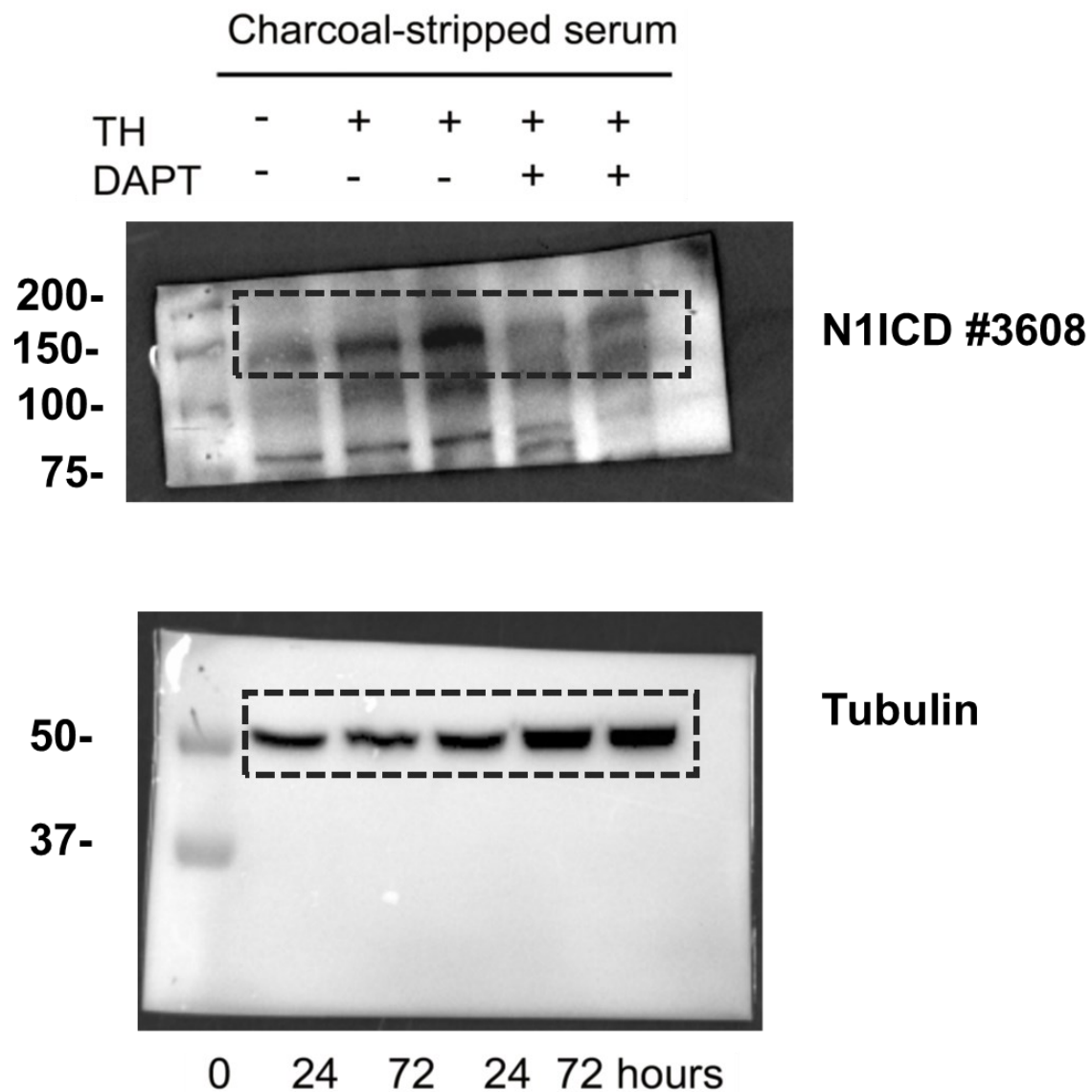

**Figure S7E**

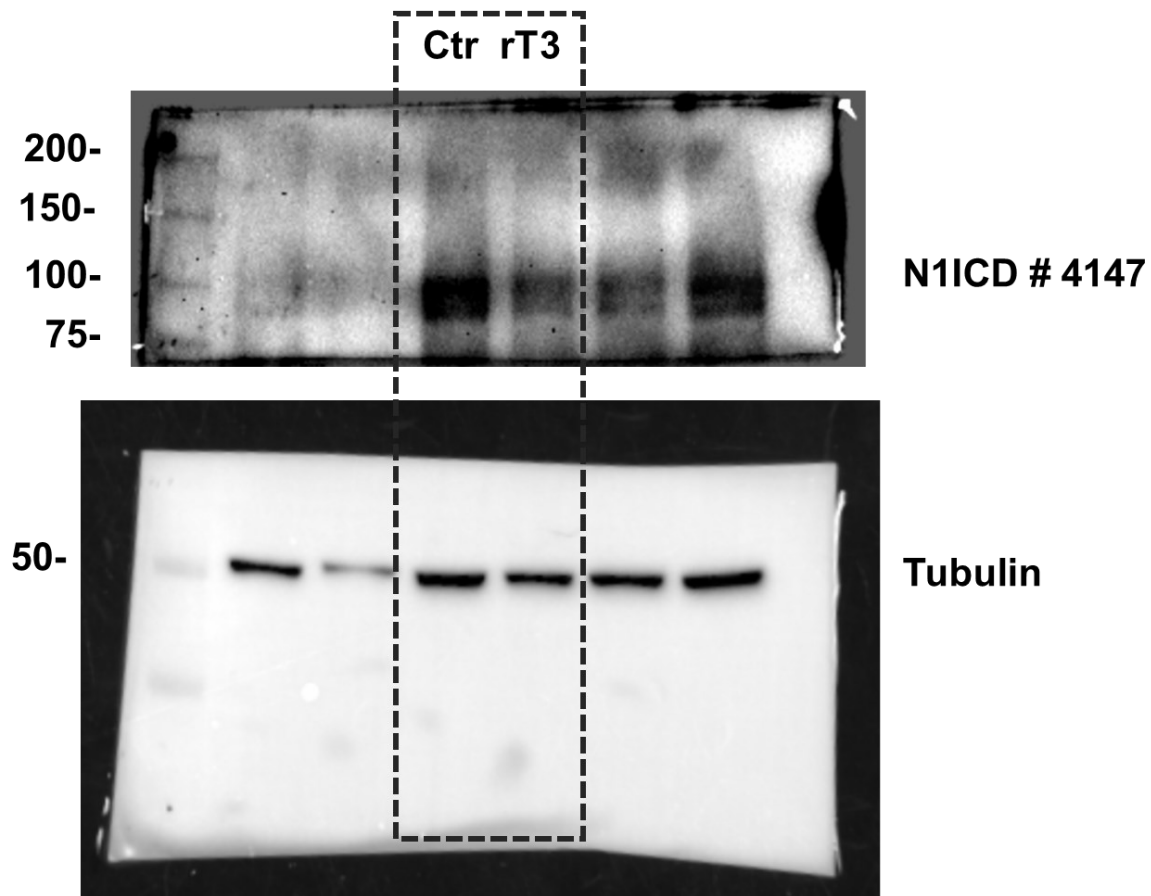

Figure S9B

Supplement: Unedited blot and gel images [file jci-136-194925-s009.pdf]
